# Cost–utility analysis of telemonitoring versus conventional hospital-based follow-up of patients with pacemakers. The NORDLAND randomized clinical trial

**DOI:** 10.1371/journal.pone.0226188

**Published:** 2020-01-29

**Authors:** Antonio Lopez-Villegas, Daniel Catalan-Matamoros, Salvador Peiro, Knut Tore Lappegard, Remedios Lopez-Liria

**Affiliations:** 1 Social Involvement of Critical and Emergency Medicine, CTS-609 Research Group, Hospital de Poniente, Almería, Spain; 2 Division of Medicine, Nordland Hospital, Bodø, Norway; 3 Institute of Clinical Medicine, Faculty of Health Sciences, University of Tromsø, Tromsø, Norway; 4 Department of Journalism and Communication, Universidad Carlos III de Madrid, Madrid, Spain; 5 Health Sciences CTS-451 Research Group, University of Almería, Almería, Spain; 6 Health Services Research Unit, FISABIO-PUBLIC HEALTH, Valencia, Spain; 7 Nursing Science, Physiotherapy and Medicine, Faculty of Health Sciences, University of Almería, Almería, Spain; University of Messina, ITALY

## Abstract

**Introduction:**

The aim of our study was to perform an economic assessment in order to check whether or not telemonitoring of users with pacemakers offers a cost-effective alternative to traditional follow-up in outpatient clinics.

**Methods:**

We used effectiveness and cost data from the NORDLAND trial, which is a controlled, randomized, non-masked clinical trial. Fifty patients were assigned to receive either telemonitoring (TM; n = 25) or conventional monitoring (CM; n = 25) and were followed up for 12 months after the implantation. A cost–utility analysis was performed in terms of additional costs per additional Quality-Adjusted Life Year (QALY) attained from the perspectives of the Norwegian National Healthcare System and patients and their caregivers.

**Results:**

Effectiveness was similar between alternatives (TM: 0.7804 [CI: 0.6864 to 0.8745] vs. CM: 0.7465 [CI: 0.6543 to 0.8387]), while cost per patient was higher in the RM group, both from the Norwegian NHS perspective (TM: €2,079.84 [CI: 0.00 to 4,610.58] vs. €271.97 [CI: 158.18 to 385.76]; p = 0.147) and including the patient/family perspective (TM: €2,295.91 [CI: 0.00 to 4,843.28] vs. CM: €430.39 [CI: 0.00 to 4,841.48]), although these large differences—mainly due to a few patients being hospitalized in the TM group, as opposed to none in the CM group—did not reach statistical significance. The Incremental Cost–Effectiveness Ratio (ICER) from the Norwegian NHS perspective (€53,345.27/QALY) and including the patient/caregiver perspective (€55,046.40/QALY), as well as the Incremental Net Benefit (INB), favors the CM alternative, albeit with very broad 95%CIs. The probabilistic analysis confirmed inconclusive results due to the wide CIs even suggesting that TM was not cost-effective in this study. Supplemental analysis excluding the hospitalization costs shows positive INBs, whereby suggesting a discrete superiority of the RM alternative if hospitalization costs were not considered, albeit also with broad CIs.

**Conclusions:**

Cost–utility analysis of TM vs. CM shows inconclusive results because of broad confidence intervals with ICER and INB figures ranging from potential savings to high costs for an additional QALY, with the majority of ICERs being above the usual NHS thresholds for coverage decisions.

**Trial registration:**

ClinicalTrials.gov NCT02237404.

## Introduction

Current guidelines call, after the period immediately following an implantation, for one to four follow-up visits per year for a standard user with a pacemaker [1,2]. Accordingly, the interval between scheduled follow-up visits after pacemaker (PM) implantation in a European survey was 12 months in 55% of centers, 6 months in 35%, and 3 months in 10% [3]. Because of increasing patient numbers [4], mainly caused by an increase in the incidence and prevalence of atrial fibrillation and chronic heart failure [5], routine follow-up of cardiac implantable electronic devices (CIEDs) contributes a significant burden to already overloaded PM consultations in terms of the time spent on human resources, as well as to patients and their caregivers because of travel costs, time, and work losses [6,7].

Telemonitoring (TM) or remote monitoring systems of pacemakers could allow access to an evaluation of the device, the patient’s clinical status, cardiovascular events, and, when required, changes in medication with a lower consumption of time and medical resources and greater comfort for patients and their caregivers [8]. Several studies have shown that TM represents a safe, effective and cost-saving way in which to significantly reduce in-office follow-up visits and lower the burden for both hospitals and patients and their caregivers [9–16]. Besides, TM has been associated with high patient acceptance and satisfaction, as well as increased adherence to programmed follow-up [17–19]. In spite of this evidence, TM of users with pacemakers is not universally adopted [20–22] and even hospitals that have incorporated this technology into routine clinical practice for other CIEDs do not routinely use it for pacemakers [23].

Cost–utility and cost–effectiveness analyses help to quantify the value of new interventions, informing both medical decision making and public policy [24,25]. Although in the last years, economic assessments of CIEDs have increased [26–34], the number of cost–utility analyses used to assess outcomes in terms of utility or quality-adjusted life years (QALYs) is scarce [35], especially in PM devices [36,37], needing more health economic studies to determine the value (the relationship between additional QALYs achieved and additional costs incurred) of systematic remote monitoring in users with pacemakers.

Furthermore, the majority of previous studies are non-randomized, which introduces the potential bias of selecting patients who are prone to the use of these technologies for some reason, whereby biasing comparisons with traditional alternatives. The Nordland study [38,39], with the accompanying economic evaluation, was designed to prospectively compare the effectiveness and cost-utility of pacemaker telemonitoring in relation to conventional hospital monitoring.

## Material and methods

### Study design and population

**Fig 1:**
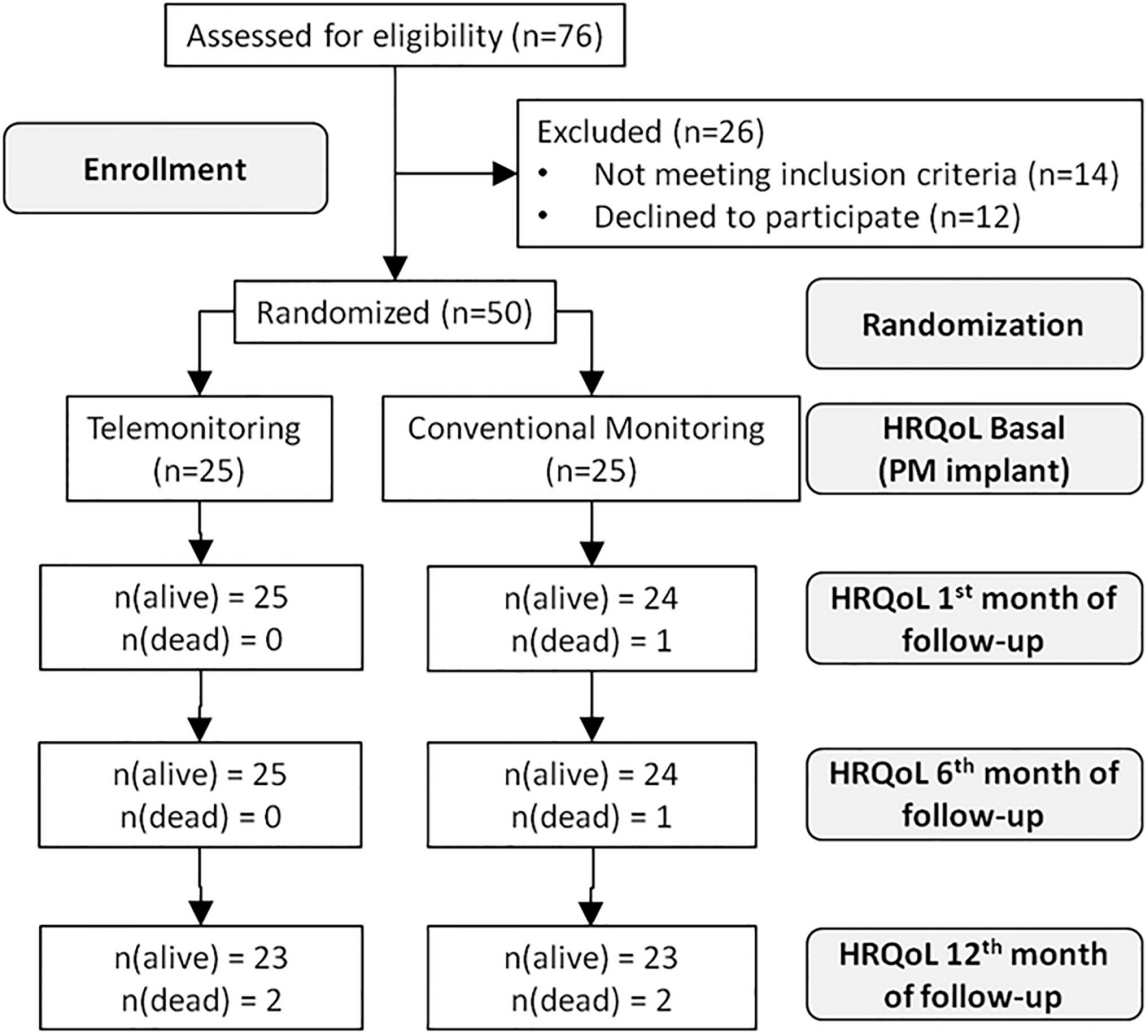
Flow diagram (CONSORT) of the study.

The NORDLAND study was designed as an open-label, 1:1, randomized, non-masked, controlled trial (S1 Appendix) to compare health-related quality of life (HRQoL) and costs with respect to the follow-up of users with an implanted PM who are assigned either telemonitoring—through electronic data transmission (intervention group, TM)—or conventional follow-up visits in the hospital (control group, CM) with 12 months’ follow-up from the date of implantation, and includes an associated cost–utility analysis. Detailed information on the trial’s design, inclusion and exclusion criteria, population characteristics, and the HRQoL preliminary results have been published elsewhere [38,39]. Briefly, between August 2014 and October 2015, 50 patients were randomized to either TM (n = 25) or CM (n = 25) in Nordland Hospital (Fig 1), which is a center with a pacemaker specialized unit serving a population of 170,000 inhabitants of Bodø (Nordland, Arctic Circle, Norway) that performs around 80–90 pacemaker implantations per year. According to their diagnosis, patients received either a single-chamber (VVIR) or a dual-chamber (DDDR) pacemaker.

Deceased patients were assigned a value of zero in the HRQoL assessments following death. HRQoL: Health-Related Quality of Life; PM: Pacemakers.

The primary outcome in the Nordland efficacy trial was concerned with the HRQoL utility weights at 12 months as assessed by the EQ5D-3L. The sample size, which was limited by the capacity of patient enrollment in the hospital, was calculated to detect a difference between groups of 0.13 points (20%) in EQ5D-3L utilities at the 12th month (the minimum clinically important difference for the EQ5D has been estimated to be between 0.05 and 0.20 according to different studies) [40], assuming a standard deviation of 0.20 with an alpha error of 0.05, as well as a power of 0.80 for a bilateral test, and a 10% of estimated follow-up losses. No adjustments for multiple comparisons were considered (S2 Appendix, shows the trial data collection forms translated into English from Norwegian).

### Ethics approval and consent to participate

The protocol was approved by the Regional Ethics Committee–REK Nord, Tromsø, Norway (Committee reference number: 2014/383/REK Nord, March 04, 2014). The study was developed in accordance with the precepts of the Declaration of Helsinki. All patients signed the corresponding informed consent prior to their enrollment (range for patient recruitment and follow-up: August 31, 2014 to November 30, 2016) and appropriate measures were taken in order to ensure data privacy. The trial protocol was registered at ClinicalTrials.gov Identifier: NCT02237404 (S1 Appendix). As the confirmation of the study registration was delayed (September 11, 2014), in order to follow the agreement with the hospital and its ethics committee, the enrolment of the first participant started twuelve days before (August 31, 2014). The authors confirm that all ongoing and related trials for this intervention are registered.

### Alternatives

#### Intervention group

Patients assigned to the remote monitoring group received either a Biotronik Estella SR-T/DR-T or a Biotronik Evia SR-T/DR-T equipped with additional storage capacity and a small RF antenna—the CardioMessenger—for wireless communication and data transmission from the implant to a wireless patient device. Home monitoring was performed through the Biotronik Home Monitoring^®^ system, which is an internet-based remote monitoring service for users with Biotronik implantable heart devices. Every night, the CardioMessenger automatically collects and transmits encrypted health information to the Biotronik service center through the use of the global network of T-Mobile and its partners (GPRS). The transmitted patient data are collected, automatically analyzed, and filtered at the Biotronik Home Monitoring Service Center, according to patients’ needs as defined by their physician. Health and system-related issues are ranked and marked in order of importance. All event and trend reports can be accessed and reviewed on a protected online platform. Furthermore, according to preset definitions, the physician can receive automatic warnings (e.g., via email or text message) concerning safety issues such as premature battery depletion, lead fracture, and so on.

#### Control group

Patients assigned to the conventional monitoring group received either one of the aforementioned Biotronik pacemakers, a St. Jude Medical Endurity SR/DR or a Sorin Reply 200 SR/DR.

### Health-related quality of life

The EuroQol five-dimension three-level questionnaire (EQ5D-3L) [41] (Norwegian version) was administered at the baseline (prior to the pacemaker implantation), at 1 month after the pacemaker’s implantation, and at 6 and 12 months of follow-up. Because EQ5D-3L Norwegian preference weights do not exist, we use Spanish weights to convert responses into single utility indices between -1 (the poorest imaginable health state) and 1 (perfect health), with death being anchored at a value of zero. There were no missing data in any of the successive surveys except for in the case of deceased patients, to whom the value of zero was assigned. QALYs were estimated as the area under the health utility curve over time through the use of linear interpolation between the weights of observations at 1, 6 and 12 months (or zero if the corresponding weight was missing due to the death of one participant) [42].

### Costs

In the main analysis, resources were measured from the perspective of the Norwegian National Healthcare System (NHS), but in secondary analysis we added a partial societal perspective including some patient/caregiver costs. The NORDLAND study included the following costs: a) physician, b) consultation room, c) ambulance, and d) hospitalizations related to the pacemaker implantation (S1 Table). All costs were provided by the Account Unit of Nordland Hospital and the standard time consumption for the two groups of follow-up was reported by the cardiology department. PM recipients and the hospitalization costs related to the device implantation were not included because are previous to the start of follow-up and independent and very similar for both alternatives. Depreciation costs for hospital equipment and instruments used in the follow-up of patients with a PM were not taken into account because they had been amortized previously. In all cases, costs were converted to euros of 2015 according to the exchange rates in that year (1 euro ≈ 9.35 Norwegian crowns). Because the follow-up period was limited to 12 months, an annual discount rate was not applied to the costs or benefits.

From the patient/caregiver perspective the following costs were taken into account: a) transportation (focusing on costs of taxis, buses, planes, trains, and patients’ private transportation), and b) the employment income loss with respect to the time spent during every in-office visit to hospital. In this case, patients’ and/or caregivers’ time spent during every visit (including travel time, waiting-room time and visit time) was considered. For caregivers, a €15/hour salary was adopted, corresponding to what a caretaker (home assistance) would earn per hour in Norway in 2015 according to the information provided by the Hospital Accounting Department. Data were collected through questionnaires administered to users during every visit to hospital or via telephone (S2 Appendix).

### Statistical analysis

First, patients’ baseline characteristics, including EQ5D-3L utility at the baseline and at month 1, and the possible differences between groups were compared with descriptive statistical analysis using mean differences for continuous variables and χ2 (replaced by the Fisher exact test when there were cells with fewer than five cases) for qualitative variables. Second, costs (visits, travel, physician time, hospitalization days, etc.), costs per patient, and outcomes including EQ5D-3L utility weights at 6 and 12 months, and QALYs were compared again using mean differences for continuous variables and χ2 (replaced by the Fisher exact test when there were cells with fewer than five cases) for qualitative variables (S2 Table, shows the statistical distribution of the variables).

Finally, incremental costs, incremental QALYs, and the incremental cost–effectiveness ratio (ICER), expressed as the incremental cost of gaining an extra QALY, were estimated. Fieller’s 95% confidence interval (CI) estimation did not yield meaningful ICER confidence limits and the resulting quadratic formula had only imaginary solutions with the Fieller confidence region covering the entire cost–effectiveness plane [43,44]. Thus, we calculate the Incremental Net Benefit (INB) with its corresponding 95%CI. The INB can be described as the increase in the number of units of effectiveness multiplied by what we are willing to pay for a unit of effectiveness (yielding the benefit of the increase in effectiveness as expressed in monetary terms) minus the increase in cost, which leaves the incremental net benefit. The INB is cost- effective if, and only if, positive (greater than zero) [45,46]. As the INB requires the specification of the willingness to pay (WTP) for a unit of effectiveness (1 QALY in our study), we use thresholds of 30,000 and 50,000 euros per additional QALY, usually considered in Europe for the incorporation of medicines or technologies into public coverage.

A probabilistic sensitivity analysis (using a parametric bootstrapping method with 10,000 simulations) was conducted in order to estimate the scatter and ellipse plots of incremental costs vs. incremental QALYs, the cost–effectiveness acceptability curves (CEACs), as well as the INB curve at different WTP thresholds with 95%CIs. Scatter and ellipse plots are figures with incremental costs on the x-axis and incremental QALYs on the y-axis, which show the distribution of ICERs generated by bootstrapping in a plane divided into four quadrants: ICERs in the upper-right quadrant imply more effectiveness with higher expenses, the lower-right quadrant would imply more effectiveness with lower expenditure (savings with improvements in outcomes, known as “dominated” situations), and the quadrants to the left would imply less effectiveness with lower (higher) expenditure or higher (lower) expense. The CEAC and the INB curve show the probability of being cost-effective for each level of WTP.

Because of a small imbalance between the groups in the number of hospital admissions during the follow-up (three in the remote monitoring group compared to none in the hospital monitoring group) and the high cost of this resource, there was a non-significant yet relevant disparity in the costs of both alternatives. Considering the possibility that this disparity could be due to chance, an unplanned analysis was performed, repeating the cost–utility analysis but excluding hospitalization costs (S3 and S4 Tables, S1 and S2 Figs).

Analyses were produced using STATA statistical software version 12 (StataCorp, College Station, Texas) (S3 Appendix, for the “do” Stata file) and the HDS online calculator for ICER, CEAC and INB probabilistic estimations (Health Decision Strategies, LLC) [47]. The CHEERS checklist for economic evaluations of health interventions has been included in the supplementary materials (S4 Appendix).

## Results

### Patient baseline characteristics

Baseline data were published previously [38,39]. The mean age of the patients was 74.8 years (CI: 71.50 to 78.18) and 48% were women, with no significant differences between both groups (Table 1). Sick sinus syndrome and atrioventricular block were the main indications for PM implantation. Dizziness (50%) and syncope (28%) were the most frequent disease manifestations, and hypertension (64%), dyslipidemia (54%) and coronary heart disease (44%) the most frequent comorbidities. No significant differences between groups were found except for diabetes mellitus (p = 0.022), which was more frequent in the CM group. As for EQ5D-3L utility, basal (0.7836; CI: 0.7193 to 0.8479) and at month1 of the PM implantation (0.7913; CI: 0.7230 to 0.8596), no significant differences between both groups were found.

**Table 1:** Patients’ clinical characteristics at baseline.

	All	Groups		p
	(n = 50)	TM (n = 25)	CM (n = 25)	
Age, mean (95%CI)	74.84 (71.50; 78.18)	73.68 (67.81; 79.55)	76.00 (72.38; 79.62)	0.491
Men (n, %)	26 (52.00)	13 (52.00)	13 (52.00)	1.000
**Pacing indication (n, %)**				
Sick sinus syndrome	24 (48.00)	12 (48.00)	12 (48.00)	0.679
Atrioventricular block	20 (40.00)	11 (44.00)	9 (36.00)	
AF with bradycardia	6 (12.00)	2 (8.00)	4 (16.00)	
**Disease manifestations (n, %)**				
Syncope	14 (28.00)	8 (32.00)	6 (24.00)	0.812
Dizziness	25 (50.00)	12 (48.00)	13 (52.00)	
Dyspnea	11 (22.00)	5 (20.00)	6 (24.00)	
**Stimulation (n, %)**				
DDDR	44 (88.00)	23 (92.00)	21 (84.00)	0.667
VVIR	6 (12.00)	2 (8.00)	4 (16.00)	
**Comorbidities (n, %)**				
Hypertension	32 (64.00)	17 (68.00)	15 (60.00)	0.556
Dyslipidemia	27 (54.00)	13 (52.00)	14 (56.00)	0.777
Coronary heart disease	22 (44.00)	8 (32.00)	14 (56.00)	0.087
Tachyarrhythmia	18 (36.00)	7 (28.00)	11 (44.00)	0.239
Diabetes mellitus	6 (12.00)	0 (0.00)	6 (24.00)	0.022
Obesity (BMI>30)	1 (2.00)	0 (0.00)	1 (4.00)	1.000
Other comorbidities	18 (36.00)	11 (44.00)	7 (28.00)	0.725
None	10 (20.00)	6 (24.00)	4 (16.00)	0.377
**Quality of life, mean (95%CI)**				
EQ5D at month 0 (pre-implantation)	0.7836 (0.7193; 0.8479)	0.7544 (0.6362; 0.8724)	0.8129 (0.7532; 0.8726)	0.366
EQ5D at 1^st^ month post-implantation	0.7913 (0.7230; 0.8596)	0.7609 (0.6484; 0.8733)	0.8216 (0.7373; 0.9061)	0.376

TM: Telemonitoring group; CM: Conventional monitoring group; CI: Confidence interval; AF: Atrial fibrillation; DDDR: Bicameral pacemaker with two electrodes placed in the atrium and in the ventricle; VVIR: Unicameral pacemaker with an electrode in the ventricle with the ability to modulate frequency of stimulation; BMI: Body mass index; EQ5D: EuroQol-5D utility weights.

### Healthcare utilization and costs

During the follow-up period (Table 2), users included in the TM group undertook a similar amount of in-office visits (1.56 [CI: 1.25 to 1.87]) but more transmissions than in the CM follow-up group (11.52 [CI: 10.08 to 12.96] vs. 1.56 [CI: 1.18 to 1.94], p<0.001) and consumed more physician time (96.60 minutes [CI: 79.64 to 113.56] vs. 46.80 minutes [CI: 35.45 to 58.15]; p<0.001). Three patients were hospitalized in the TM group (in all cases related to the pacemakers’ functioning) compared to none in the CM group (2.28 hospitalization days vs. none; p = 0.142). The distance from home to hospital was higher in the TM group (93.08 Km [CI: 41.99 to 144.16] vs. 56.68 Km [CI: 27.83 to 85.53]), as was the time spent by patients on travel and visits (379.20 minutes [CI: 236.98 to 521.42] vs. 307.02 minutes [CI: 222.21 to 392.19]), both variables were not statistically significant not statistically significant in both variables.

**Table 2:** Cost inputs, costs per patient year, and quality of life outcomes.

	All	Groups		*p*
	(n = 50)	TM (n = 25)	CM (n = 25)	
***Cost inputs*, *mean (95%CI)***				
In-office visits, patient/year	1.56 (1.25; 1.87)	1.56 (1.04; 2.08)	1.56 (1.18; 1.94)	*1*.*000*
PM transmission, patient/year	6.54 (4.94; 8.14)	11.52 (10.08; 12.96)	1.56 (1.18; 1.94)	*<0*.*001*
Physician time, min/patient	71.70 (59.54; 83.86)	96.60 (79.64; 113.56)	46.80 (35.45; 58.15)	*<0*.*001*
Hospitalization days	1.14 (0.00; 2.70)	2.28 (0.00; 5.43)	0.00 (0.00; 0.00)	*0*.*142*
Distance home/hosp., Km	74.88 (46.13; 103.63)	93.08 (41.99; 144.16)	56.68 (27.83; 85.53)	*0*.*206*
Patients’ time (travel & visits)	343.20 (262.70; 423.70)	379.20 (236.98; 521.42)	307.02 (222.21; 392.19)	*0*.*374*
***NHS costs (€ 2015)*, *mean (95%CI)***				
Physician	56.71 (47.10; 66.33)	76.41 (62.99; 89.83)	37.02 (28.04; 45.99)	*<0*.*001*
Consultation room	29.04 (24.11; 33.96)	39.12 (32.25; 45.99)	18.95 (14.36; 23.55)	*<0*.*001*
Hospitalization	904.16 (0.00; 2,137.90)	1,808.31 (0.00; 4,311.46)	0.00 (0.00; 0.00)	*0*.*142*
Ambulance transport	186.00 (119.53; 252.47)	156.00 (75.11; 236.89)	216.00 (105.70; 326.30)	*0*.*370*
Total NHS costs	1,175.90 (0.00; 2,423.84)	2,079.84 (0.00; 4,610.58)	271.97 (158.18; 385.76)	*0*.*147*
***Patient/family costs (€ 2015)*, *mean (95%CI)***				
Patient travel % waiting costs	42.40 (19.11; 65.69)	58.86 (14.14; 105.58)	24.94 (13.19; 36.69)	0.133
Accompanying person costs	85.80 (65.67; 105.93)	94.80 (59.24; 130.36)	76.80 (55.55; 98.05)	0.374
Other transport costs	59.04 (18.64; 99.44)	61.41 (0.00; 124.98)	56.68 (2.03; 111.33)	0.907
Total patient costs	187.24 (117.34; 257.15)	216.07 (83.41; 348.73)	158.42 (102.26; 214.58)	0.413
***Total (NHS + patient family) costs (€ 2015)*, *mean (95%CI)***				
Total costs	1,363.15 (105.36; 2,620.93)	2,295.91 (0.00; 4,843.28)	430.39 (0.00; 4,841.48)	0.138
***Outcomes***				
Hospitalizations n (%); (95%CI)	3 (6.00) (1.25; 16.55)	3 (12.00) (2.55; 31.22)	0 (0.00) (0.00; 13.72)	0.074
Deaths n (%); (95%CI)	4 (8.00) (2.22; 19.23)	3 (12.00) (2.55; 31.22)	1 (4.00) (0.10; 20.35)	0.297
EQ5D at month 6 (mean, 95%CI)	0.7533 (0.6670; 0.8396)	0.8158 (0.7003; 0.9313)	0.6907 (0.5590; 0.8224)	0.147
EQ5D at month 12 (mean, 95%CI)	0.7561 (0.6714; 0.8407)	0.7291 (0.6014; 0.8569)	0.7830 (0.6634; 0.9026)	0.528
QALYs (mean, 95%CI)	0.7635 (0.6998; 0.8271)	0.7804 (0.6864; 0.8745)	0.7465 (0.6543; 0.8387)	0.598

TM: Telemonitoring group; CM: Conventional monitoring group; SD: Standard deviation; PM: Pacemaker; NHS: National Health System; EQ5D: EuroQol 5D utilities; QALYs: Quality-adjusted life years.

Accordingly, physician costs (€76.41 [CI: 62.99 to 89.83] vs. €37.02 [CI: 28.04 to 45.99], p<0.001); consultation room costs (€39.12 [CI: 32.25 to 45.99] vs. €18.95 [CI: 14.36 to 23.55], p<0.001); and hospitalization costs (€1,808.31 [CI: 0.00 to 4,311.46] vs. €0.00, p = 0.142) were higher in the telemonitoring group, but differences were not significant for the latter. Overall, total costs from the perspective of the NHS were higher for the TM group (€2,079.84 [CI: 0.00 to 4,610.58] vs. €271.97 [CI: 158.18 to 385.76]; p = 0.147), especially due to the cost of hospital admissions, although these large differences did not reach statistical significance.

Total patient and caregiver costs were lower in the conventional monitoring group (€158.42 [CI: 102.26 to 214.58]) than in the TM group (€213.99 [CI: 83.41 to 348.73]), albeit not statistically significant. Overall, total costs per patient were higher—albeit not statistically significant—in the TM group (€2,295.91 [CI: 0.00 to 4,843.28]) than in the CM group (€430.39 [CI: 0.00 to 4,841.48]), mainly due to the cost related to hospitalizations.

There were three (12% [CI: 2.55 to 31.22]) hospitalizations and three deaths (12% [CI: 2.55 to 31.22]) in the RM group compared to none (0% [CI: 0.00 to 13.72]) and one (4% [CI: 0.10 to 20.35]) in the CM group, without significant differences between groups in both variables. At 6 months the mean EQ5D-3L scores were 0.8158 (CI: 0.7003 to 0.9313) in the TM group and 0.6907 (CI: 0.5590 to 0.8224) in the CM group, and at the end of the follow-up period the mean EQ5D-3L scores were 0.7291 (CI: 0.6014 to 0.8569) and 0.7830 (CI: 0.6634 to 0.9026) respectively, also without significant differences. Overall, patients in the TM group obtained 0.7804 (CI: 0.6864 to 0.8745) QALYs during the follow-up compared to 0.7465 (CI: 0.6543 to 0.8387) QALYs for patients in the CM group, with a non-significant difference of 0.0339 (CI: -0.1622 to 0.0944) QALYs favoring the TM group.

### Cost–utility analysis

Mean NHS costs per QALY (TM: €2,623.60 [CI: 0.00 to 6,001.63] vs. CM: €416.85 [CI: 227.12 to 606.59]; p = 0.171) and mean total costs per QALY (including patient/caregiver costs) (TM: €2,874.90 [CI: 0.00 to 6,166.53] vs. CM: €742.86 [CI: 339.30 to 1,146.41]; p = 0.191) were higher in the TM group (Table 3), but the differences did not reach statistical significance. Incremental costs per patient included in the TM vs. CM group constituted €1,807.87 (CI: -646.99 to 4,262.73) from the perspective of the NHS and €1,865.52 (CI: -608 to 4,335.25) including patient/family costs. Because incremental QALYs per patient using TM vs. CM were minimal, the mean ICER amounted to €53,345.27 from the perspective of the NHS or €55,046.40 including patient/caregiver costs, through the incremental QALY gained. All estimated INBs were below zero, both using thresholds of €30,000 or €50,000 and from the perspective of the NHS or including patient/family costs, albeit in all cases with wide confidence limits that far exceeded zero at its upper limit.

**Table 3:** Incremental costs per QALY (cost–utility analysis) of TM vs. CM perspective.

	NHS costs	Total costs
Incremental costs per patient	1,807.87 (-646.99; 4,262.73)	1,865.52 (-608; 4,335.25)
Incremental QALYs per patient	0.0339 (-0.0937; 0.1615)	0.0339 (-0.0937; 0.1615)
Mean ICER (€)	53,345.27	55,046.40
Mean INB_(WTP30000)_(€)	-791.17 (5,420.81; 3,838.47)	-848.82 (-5,478.22; 3,784.72)
Mean INB_(WTP50000)_(€)	-113.37 (-7,041.43; 6,814.70)	-171.02 (-7,095.04; 6,757.14)

TM: Telemonitoring group; CM: Conventional monitoring group; QALY: Quality-adjusted life year; NHS: National Health System; ICER: Incremental cost–effectiveness ratio; INB: Incremental net benefit; WTP: Willingness to pay.

**Fig 2:**
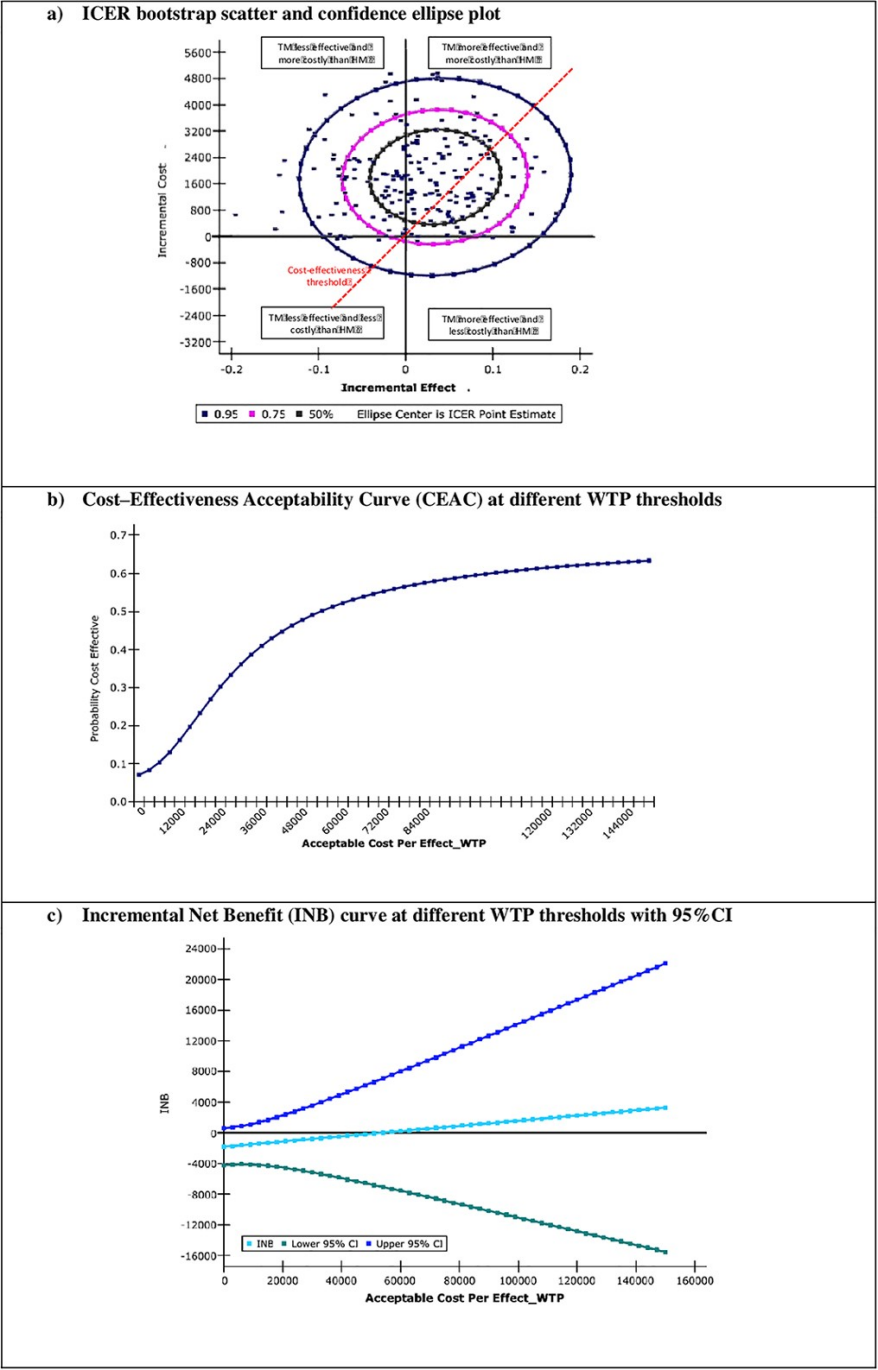
Incremental costs per QALY from NHS perspective; probabilistic sensitivity analysis. NHS: National Health System; TM: Telemonitoring; CM: Conventional Monitoring group; QALY: Quality Adjusted Life Year; ICER: Incremental Cost-effectivenes Ratio; WTP: Willingness to Pay.

In the probabilistic analysis from the perspective of the NHS, and because of the small number of hospitalized patients with high costs, the 95% of ICERs generated by the bootstrapping simulation (Fig 2a, scatter and ellipse plots) show a higher probability that the ICERs will be located in the areas that indicate a higher cost of telemonitoring with greater effectiveness (61% of the ICERs are located in the upper-right quadrant of the cost–effectiveness plane) or a higher cost with less effectiveness (37% of the ICERs are located in the upper-left quadrant), with the probability of lower costs of telemonitoring (lower quadrants) being remote. The CEAC (Fig 2b) shows a probability of around 35% that telemonitoring would be cost-effective for a threshold of €30,000 per additional QALY, with something below 50% for a threshold of €50,000. The INB curve (Fig 2c) shows concordant results, with INBs being less than zero from thresholds lower than €50,000 for additional QALYs earned. Furthermore, the INB curve also shows the wide range of 95% confidence intervals.

The probabilistic analysis including the patient/caregiver perspective shows very similar results (S3–S5 Figs). The unplanned analysis excluding hospitalization costs (S3 and S4 Tables, S2 and S5 Figs) shows mean costs per QALY which are practically the same between alternatives from the perspective of the NHS with positive INBs at €30,000 and €50,000 thresholds, whereby suggesting a discrete superiority of the TM alternative, although in both cases the 95%CIs are broad and include negative values.

## Discussion

In this randomized study, with 12 months of follow-up after PM implantation, both alternatives were similar in terms of QALYs gained, but incremental costs per QALY were higher in the TM group than in the CM group, both from the perspective of the Norwegian NHS and including patient and caregiver costs. Although differences did not reach statistical significance, probably due to the small sample size, the ICER was somewhat above €50,000 for additional QALYs, which is one of the thresholds frequently used in Europe to include new technologies in public assurance coverage. These results were very dependent on the consideration of a few cases from the RM group who had episodes of hospitalization during the follow-up period. The probabilistic analysis showed wide confidence intervals and the unplanned analysis excluding hospitalization costs demonstrated a situation of practical equivalence between alternatives with a discrete advantage in cost-effectiveness for TM.

These results are consistent with the results of bivariate analysis that found no relevant differences in any outcome—including QALYs—or in costs (except for hospitalization), but contrast with those found in studies on costs and cost-effectiveness in other types of CIEDs (such as implantable cardioverter defibrillators or cardiac resynchronization therapies) in which there were significant differences—albeit not always relevant—found in utilization and costs favoring the telemonitoring group with equal or higher effectiveness [48–56], whereby suggesting that telemonitoring is a cost-effective (or dominant) alternative compared to conventional follow-up in hospital, as well as with the results of economic PM studies comparing both types of follow-up, suggesting lower utilization and costs, as well as cost-effective indices favoring remote monitoring alternatives [57].

The reasons for these discrepancies are diverse. First, the remarkable heterogeneity of the alternatives in comparison to different hospitals in both the TM group (which may include different devices, technologies, numbers of transmissions, top or bottom monitoring, configuration of alerts, hospital visit scheduling, etc.) and the “usual care” group can be extraordinarily different between hospitals. Second, costs included in the different studies are highly variable and, in general, partial (sometimes being limited to transportation or visits to the heart rate unit), as are the measures of effectiveness or the construction of utilities. Third, published cost–effectiveness studies usually do not include an assessment of uncertainty (probabilistic sensitivity analysis) in the estimation of the ICER or INB, with it being probable (because of the scarce—and almost always non-significant—differences in utility) that broad confidence intervals are offered with figures ranging from potential savings to increased spending. Finally, several previous studies have been non-randomized, which may have affected the composition of the study groups.

It is foreseeable that the majority of figures included in our study are above the cost–effectiveness thresholds usually accepted in Europe (between 30,000 and 50,000 additional euros for an additional QALY). It seems reasonable to expect that the efficiency of pacemakers’ telemonitoring does not depend so much on the technology itself as on the organizational model for providing these services. The incorporation of this technological innovation without the corresponding organizational change could lead to configuring it as an incremental service (with processes being added to a previous monitoring protocol) rather than as a substitute service with respect to conventional hospital-based monitoring, with no savings except if there were a notable increase in effectiveness (but the majority of studies with pacemakers—not with other CIEDs—tend to suggest equivalence in effectiveness, safety, and quality of life). A radical organizational change, however, could lead to significant reductions in costs, as suggested by some observational studies with remote monitoring follow-up practically replacing in-clinic device checks [58]. In this area, and while it is assumed that one of the main advantages of RM would be the reduction of travel and costs for patients and their families (especially in dispersed territories such as Nordland, in the Northern Norway region), the similarity of travel costs (and total costs for patients and families) between both alternatives in our study is surprising and reinforces the idea that the introduction of remote monitoring in this case was not accompanied by an organizational change so as to take advantage of this new technology.

This cost–utility analysis has some methodological weaknesses and strengths. First, its power was insufficient to detect differences between the two strategies and, additionally, rendered the results very sensitive to the impact of infrequent events due to chance. The small sample size, which was a limitation derived from the hospital’s own activity and the time available for recruitment, generated results that were subject to high uncertainty with wide confidence intervals. In any case, and considering the differences found (excluding the hospital costs), even a very large study would be unlikely to generate different results. Second, it is a non-masked study wherein both the patients and the research team knew the type of follow-up assigned which could influence their behavior. Its open-label character allows the appearance of certain information biases such as the Hawthorne effect (patients modifying their behavior in response to their awareness of being observed), social desirability bias (patients overreporting positive behaviors or underreporting undesirable ones), performance bias (physicians modifying their behavior), detection bias (outcome information being collected differently between groups), and so on. Third, the costs considered compose only a proportion of the healthcare costs, including other aspects such as medication, diagnostic tests, visits to primary care, emergencies, those related to the underlying disease, and so on, and probably do not include all costs from the social perspective. Although in this study—as in almost all analogous studies—it has been assumed that these costs are not differential between alternatives, it is possible that the type of follow-up affects some of them. Fourth, because of the lack of Norwegian EQ5D weights, we use EQ5D-3L Spanish weights. In theory, between-country differences in preference values for health states can lead to between-country differences in utility scores, whereby affecting the study results. However, between-country cross-sectional studies show an important similarity among EQ5D-3L weights (generally below 0.1) and great consistency between the different health states [59]. Finally, because it is a single-center study, generalization to other centers (which may have other follow-up protocols) should be assessed with great caution.

## Conclusions

In summary, this study provided evidence showing that 12 months after pacemaker implantation, health-related quality of life was similar between groups of RM and conventional follow-up in hospital, while follow-up costs were higher in the RM group, mainly due to differences in hospital admissions. The main cost–utility analysis shows broad confidence intervals with ICERs and INBs ranging from potential savings to high costs for an additional QALY, with the majority of ICERs being above the usual NHS thresholds for coverage decisions.

## Supporting information

S1 Appendix Trial protocol. (PDF)

S2 Appendix Data collection forms (English version). (PDF)

S3 Appendix Do file (STATA). (PDF)

S4 Appendix Consolidated Health economic evaluation reporting standards (cheers) statement. (PDF)

S1 Table Costs included in the economic evaluation. (PDF)

S2 Table Statistical distribution of study variables. (PDF)

S3 Table Cost per patient-year. (PDF)

S4 Table Incremental costs per QALY. (PDF)

S1 Fig ICER bootstrap scatter and confidence ellipse Plot. (PDF)

S2 Fig Incremental Net Benefit (INB) curve at different WTP thresholds with 95%CI. (PDF)

S3 Fig Incremental net benefit. (PDF)

S4 Fig Cost-effectiveness acceptability curve. (PDF)

S5 Fig Incremental net benefit curve at different WTP thresholds with 95% CI. (PDF)
