# Supplementary material for: Cost–utility analysis of telemonitoring versus conventional hospital-based follow-up of patients with pacemakers. The NORDLAND randomized clinical trial
Source: PLoS One. 2020 Jan 29;15(1):e0226188. doi: 10.1371/journal.pone.0226188 (PMC6988929; doi:10.1371/journal.pone.0226188)
Supplement: S1 Appendix — (PDF) [file pone.0226188.s001.pdf]

1. SUMMARY OF THE PROPOSAL

TITLE OF THE PROJECT: **Sustainability of cardiology services: economic evaluation in communication and remote monitoring of people with pacemakers.**

Daniel Catalán-Matamoros[1], Knut Tore Lappegård[2], Salvador Peiró-Moreno[3], Remedios López-Liria[4], Antonio López-Villegas[4].

Partner Institutions

1. Madrid University Carlos III, Madrid. Spain.
2. University of Tromsø, Tromsø. Norway
3. Foundation for the Promotion of Health and Biomedical Research the Valencia Community. Valencia. Spain.
4. University of Almeria, Almería. Spain.

SUMARY OF THE PROJECT

Introduction

This project is the result of several months of preparatory work by a multidisciplinary team of professionals (physicians, psychologists, communication specialists, health economics and management/administration experts) from various institutions such as Universities, Hospitals and Research Centres of Madrid, Valencia and Almeria in Spain and the Norwegian cities of Tromsø and Bodø.

The financial crisis that most Western countries are suffering has dramatically cut down the public budgets for the health sector. This reduction has forced healthcare managers to improve efficiency in current planning systems, managerial strategies and better cost-effective allocation of technologies and resources.

Mathematics, its different assessment models and temporal simulation, have helped to assess and predict the evolution of healthcare technology in the short, medium and long term. The Economic Evaluation of Healthcare Technologies is the main mathematical tool used globally in the evaluation of the efficiency of a particular technology. This tool may help to assess the relationship of costs and the health outcomes, providing relevant information to the healthcare system to consider whether a technology should be funded or not.

Cardiovascular Diseases are a major cause of global morbidity and mortality, being responsible according of 16.7 million deaths around the world (30% of overall mortality) to the World Health Organization. The 25.75% (4.3 million) of these deaths occur in Europe and the 50% in the European Union, with an estimated cost of 192 billion € / year. Previous studies have indicated that of the total cost, 57% is due to health care costs, 21% to productivity losses and 22% to the informal care of people with cardiovascular diseases.

In the field of Cardiology, Telemedicine allows consultations with patients through monitoring systems and remote communication analyzing the ongoing heart rates of people with pacemakers, implantable cardioverter defibrillators, cardiac resynchronization therapy and subcutaneous Holter. The use of remote monitoring may save time and efforts to both healthcare professionals and patients, including their informal caregivers, reducing the number of follow up visits to the hospital and reducing the associated costs with patient follow-up, which will help to improve sustainability of healthcare services.

Since 2001 that the first pacemaker of remote monitoring was implanted in Europe, more than 300,000 pacemaker have been implanted around the world. Despite this sharp expansion, the scientific evidence on economic evaluations of pacemaker with remote monitoring is very limited, and in our knowledge, studies including informal costs have not been conducted. Most of the studies have just focused on implantable cardioverter defibrillators and cardiac resynchronization therapy.

Objective

Analyze the new communication systems and remote monitoring of people with pacemaker implantation in relation to the sustainability of cardiology services in our current society.

Methodology

Effectiveness Analysis

Open trial, without masking but with randomization. The study sample (n=38) will be composed by all patients who have been implanted with a pacemaker in the Nordland Hospital (Bodø) between 15/08/2014 and 14/03/2015 with a follow up of 6 months. At the same time 2 groups will be formed according to the type of technology installed:

a) Intervention group: composed of 19 patients with a remote monitored (RM) pacemaker (PM).
b) Control group: formed by 19 patients with a hospital monitored (HM) PM.

During the monitoring period, the same parameters were analyzed in both groups at 3 different times (pre-implant and at months 1 and 6 post-implantation). Instruments are:

a) Clinical History.
b) Generic HRQOL Questionnaire, EuroQol-5D (EQ-5D).
c) Specific Questionnaire on HRQOL in patients with CVD, Duke Activity Index.

Cost Analysis

Qualitative and quantitative design. Costs will be estimated from both the healthcare perspective and the social perspective.

Identification and quantification of healthcare costs:

a) In-depth interviews with the heads of the Cardiology Units and Accounting Department.
b) Clinical History.
c) Information provided by the Accounting Unit.

Identification and quantification of informal costs:

a) Principal Caregiver Questionnaire.
b) Satisfaction survey.
c) Interviews with patients and families.

Economic Impact:

a) Construction of a decision tree, which allows to know the alternatives, events and results of applying both types of monitoring.
b) Mathematical simulation through cohorts: Markov Model.
c) Explore how communication and remote monitoring of pacemaker can provide added value to the sustainability of the current healthcare system.

Expected results

1) Patients' perception regarding to their own health at different times: before and after pacemaker implantation.
2) Costs associated with the monitoring of patients with pacemakers.
3) Economic impact of remote monitoring of pacemaker versus hospital monitoring along time.

Key words: Pacemakers, Telemedicine, Remote Monitoring, Cost-benefit and Cost-effectiveness.

2. DESCRIPTION OF THE PROJECT/ DESCRIPCIÓN DEL PROYECTO

Aims

General objective 1: Analyze the effectiveness of remote monitoring (RM) in patients with pacemaker (PM) implant compared to the hospital mode.

Specific Objectives

1) To analyse the clinical and socio-demographic characteristics of both groups.
2) To assess the health-related quality of life (HRQoL) and patient satisfaction at different times pre and post implant.

General objective 2: Analyze the costs of remote monitoring (RM) versus hospital monitoring (HM), of PM cardiac patients.

Specific Objectives

1) To identify and assess the direct and indirect health costs resulting from the RM system of patients with PM implant and that applied in the hospital.
2) To analyse the costs that both types of monitoring incur in relation to informal care needed by people with pacemaker implants.

General objective 3: To assess the impact and cost effectiveness in the medium and long-term of RM vs hospital monitoring (HM): Markov's mathematical model.

Specific Objectives

1) To build a decision tree that allows us to know the alternatives, events and results of applying both types of monitoring.
2) To carry out a mathematical simulation through cohorts.

To explore how communication and the remote monitoring of cardiac devices can add value to the sustainability of the current healthcare system.

Originality and innovative aspects of the project

It is an original study since the literature on cost-effectiveness in the remote monitoring of PM are very limited[21]. Most studies have focused on other cardiac devices such as automatic implantable defibrillator (AID)[16-18] and cardiac resynchronization therapy (CRT) [19.20]

Differentiating and novel aspects will involve:

1. The analysis of a new monitoring in patients with a pacemaker.
2. Assessing the Quality of Life Related to Health and satisfaction of patients with PM.
3. The identification and assessment of both health care costs and costs associated with informal care (including those related to dependency).

The estimation of the economic impact of RM over time compared with the hospital pacemaker mode.

How shall it contribute to the advance of the knowledge

This study will show the level of efficiency for both society and for the sustainability of the public health services that this new technology has when applied to pacemakers, and also allows us to determine the degree of savings for both the National Health System and to patients and their environment.

The results will be relevant for political decision-making, health service managers, users and providers of heart technologies both in Spain and Norway, and other countries with similar socio-health characteristics. In addition to this study, due to its innovative nature in relation to services for monitoring and remote communication, it may also be useful for other countries, where patient access to hospitals poses a significant effort due to the orography, means of transport used, proximity to the hospital, economic capacity, etc. Note that Norway is a country that has a wide geographical dispersion of the population, and therefore, these systems become even more relevant in order to bring health to the entire population thus ensuring the sustainability of the health system.

Background, conceptual framework, open questions and approaches, including the justification of the importance of the issue to be faced

Economic Evaluation of Health Technologies

Healthcare Technology is understood as the set of drugs, devices and medical or surgical procedures used in healthcare, and the organizational and support systems within which such care is encouraged[22]. Health technology is intended for the prevention, diagnosis, treatment and rehabilitation of specific clinical conditions and to improve the quality of life of individuals and society [23].

Despite the economic context of reducing the weight of the public sector in most Western countries, health spending has been steadily growing in recent years [24].

Since the creation of the Agencies of Health Technology Assessment (AHTA) in Europe in the last decade of the twentieth century, the National Institute for Health and Clinical Excellence (NICE) in the UK stands out. Mathematics and its different temporal assessment and simulation models have helped to assess and predict the evolution of a health technology in the short, medium and long term[2-5].

The Economic Evaluation of Health Technologies (EEHT) is the main standardized mathematical tool used globally in evaluating the efficiency of a particular technology. This instrument will help to assess the relationship of costs invested in health outcomes obtained[6], helping different national health systems to consider whether a technology should be funded or not. The techniques used in an economic evaluation are: cost minimization, cost-effectiveness analysis, cost-benefit analysis and cost-utility analysis[2.3].

Cardiac Pacemakers

A cardiac pacemaker is a device that senses when the heart is beating irregularly. The PM works by sending a constant signal to the heart, so that it beats correctly[25].

The most frequent electrocardiographic abnormalities before implantation are atrioventricular block (AVB) with 56.0%, sick sinus syndrome (SSS) with 19.9%, atrial fibrillation or flutter with slow ventricular response (16.4 %) and intraventricular conduction disorder (5.5%)[21].

Between implants and replacements, in Spain in 2012, 34,919 PMs were implanted (745.8 PM / million inhabitants)[21], which contrasts with the 2,333 PM implanted in Norway in 2009 (489 PM/million inhabitants)[26]. The mean age of implanted patients is over 70 years in both cases. Due to the aging population and the increase in life expectancy, the incidence of degenerative diseases increases, which is the cause of most implants. Males represent 57.1% of PMs implanted in Spain[21], and that percentage reduces to 55.7% in the case of Norway[26].

Hospital Monitoring (HM) vs Remote Monitoring (RM)

HM means check-ups that patients with PM have at the specialist's office, where two functions are mainly performed[10.25]:

1) Control problems that patients have with their heart.
2) Assess the proper functioning of the cardiac device.

As a solution to the complexity of monitoring, in recent years new technology has developed that is capable of RM in patients implanted with PMs.

The cardiologist has free access to information from the cardiac device, at any time of day. And if at any given moment, the specialist detects any anomaly they could alert the patient (phone, email, etc.) so that they come to the hospital.

Table 1. Advantages and disadvantages of RM[10-13,27,28] .

Advantages	Disadvantages
Ease of use, depending on the type of PM implanted.	Increased staff and budget due to the large volume of information generated.
Telematic registration and assessment of the correct functioning of the PM.	It may adversely affect the patient's attitude, since it can be falsely appreciated as an alternative route for absolute and immediate access to care.
Rapid detection of changes in both heart rate and the operation of the device.	Alerts and other information resulting from the RM can alter the perception that the receiving device has on health.
Saves time and effort for both health professionals and patients and their companions.	
Increased care for older patients, and more complex.	
Decreased costs associated with monitoring the patient.	

Justification

Cardiovascular diseases are a major cause of mortality and morbidity.

In 2008, non-communicable chronic diseases in people 60 and older, accounted for the greatest burden of disease attributed 95.5% of disability-adjusted life year (DALY), corresponding to 20.4% of cardiovascular diseases (CVD)[29].

Cardiovascular diseases are a major cause of global morbidity and mortality, and are responsible, according to the World Health Organization, for about 16.7 million deaths worldwide (30% of overall mortality)[29]. 25.75% (4.3 million) of these deaths occur in Europe, and 50% in the European Union (EU), which have an estimated cost of 192 billion €/year. Previous studies[7] have indicated that the total cost, 57% is due to health care costs, 21% to productivity losses and 22% to the informal care of people with CVD.

Work overload in pacemaker consultations

There is a work overload in pacemaker consultations due to an exponential increase in the number of implanted cardiac devices and the increase in technological complexity thereof and the software to manage them.

The implantation of a PM needs monitoring (as well as the implanted patient), in which various electrical parameters will be analysed and adjusted in order to check for correct operation, increased longevity, improved quality of life of patients and prolonged survival[12].

Insufficient research

Despite the sharp expansion, the scientific evidence on economic evaluations in the remote monitoring of PM is very limited[4.15] and no studies that include informal costs have been found. Most existing studies have focused on AID[16-18] and CRT[19.20].

Interest

To the best of our knowledge this would be a pioneering study, since research to date has focused on the analysis of various cardiac devices, which is different to the purpose of our project. A distinctive and novel feature will be the ability to identify and evaluate the costs associated with informal care - including those related to dependence - in patients with pacemaker implants.

This study will show us the cost-effective potential that this new technology has applied to PMs and, if there is an ability for saving for both the National Health System and patients.

Describe de expected results and the expected impact of such results

Effectiveness Study

The review of *Medical Records* will provide us with the information necessary for the evaluation of sociodemographic and clinical variables, and those corresponding to the functioning of the PM.

The administration of the *EuroQol-5D (EQ-5D) and* Duke Activity Status Index *questionnaires,* will show the *patients' perception regarding the quality of life related to health at different pre and post PM implant times*.

Study Costs

The *in-depth interviews* of those responsible for the departments of Cardiology and Accounting both hospitals, coupled with the review of the *Medical Records* - throughout the study period - will allow us to *identify the direct/healthcare costs*.

Access to the data provided by the accounting department will provide the healthcare costs associated with the monitoring of such implants.

To quantify *the informal costs* associated with this type of care, the following instruments will be used:

a) Survey on Disability, Personal Autonomy and Dependency to informal caregivers in month 6 (post-implant).
b) *Satisfaction Survey* on patients at month 6 (post-implant), which include aspects related to displacement, informal caregivers and hospital visits.

Economic Impact

It will evaluate the impact and cost-effectiveness in the medium and long term compared to HM and RM. The tools we will use are:

1) To build a decision tree that allows us to know the alternatives, events and results of applying both types of monitoring.

2) Carry out a mathematical simulation through cohorts.

Explore how communication and the RM of cardiac devices can add value to the sustainability of the current healthcare system.

Describe how the planned cooperation shall contribute to the success of the project, and how will it benefit each involved part

Cooperation between different groups will enable the exchange of knowledge. It will assess whether the use of the same technology applied in two different healthcare systems provides similar data on effectiveness, costs, and cost-effective impact in the medium and long term.

Working together will strengthen links between researchers in both countries, providing the possibility of working in the future together.

The participation of each institution will benefit the project as follows:

- Madrid University Carlos III (Madrid, Spain): contribution of knowledge necessary for effective communication in the field of health, experience in management and coordination of international projects.
- University of Tromsø: access to patients with PM in Nordland Hospital (Bodø, Norway), and experience in projects on patients with PM in small and medium sized diversified centers.
- Foundation for the Promotion of Health and Biomedical Research the Valencia Community. (Valencia, Spain): an international reference in Economic Evaluation of Health Technologies and experience in developing international high-impact projects in the field of health economics.
- University of Almería (Almería, Spain): access to pacemaker patients in the Hospital de Poniente, conducting doctoral thesis based on the Economic Evaluation of RM pacemakers, development of projects based on home care of patients with different diseases.

The institutions described above form a key multidisciplinary team that brings together all areas included in this project.

Describe the working plan and working methods, new methodologies or techniques to be used or created. Provide a plan of visits and exchanges, names and host and sending institutions.

Working plan

Month 1:

- Meeting of all the research team from all institutions via videoconference.
- Meeting with the Norwegian research team:
  - University of Tronsø:
    - Partner and co-director of the project.
    - Pre-doctoral student.
  - Nordland Hospital (Bodø):
    - Head of Cardiology Department (health variables).
    - Head of Accounting Department (economic variables).

Month 1-13:

- Data collection.
- Start writing the manuscript.
- Analysis of the results obtained.

Month 13:

- Finalization administrative issues with University of Tronsø and Nordland Hospital in Bodø.

Currently the project is being developed at the Hospital de Poniente (Almería, Spain). Ended data collection period in both hospitals, the results will be compared.

The project format has been adapted in response to the aspects mentioned above in the field of economic evaluation in cardiology and shall cover the 3 general objectives presented below.

General objective 1: To analyse the effectiveness of RM in patients with PM implants compared to the hospital mode.

Design

Open-label, unblinded without randomization.

Participants

Nordland Hospital (Bodø) has a catchment population of approximately 180,000 inhabitants. Between 80-90 PM implants/year [26]. The sample of our study was calculated taking into account the following parameters:

Margin of error that we would be willing to accept (%)	5
Confidence level (%)	95
Population size (PM/year)	90
Heterogeneity level (%)	50
Sample size (PM / year) recommended	74
Sample size (PM / 6 months) recommended	37

The study sample (n = 38) will consist of all patients who have been implanted with a PM in the Nordland Hospital (Bodø) between 15/08/2014 and 14/03/2015, with follow up 6 months later. In turn, 2 groups will be formed according to the type of implanted technology:

a) Intervention group: composed of 19 patients who have been implanted with a RM PM.
b) Control group: Formed by 19 patients with implanted a HM PM.

Prior to implantation, the cardiologist will explain the features of both types of patient PM monitoring (including advantages and disadvantages), and depending on their personal characteristics and access to the telephone network, the type of pacemaker they want implanted must be chosen. The choice will be given in all cases, except for: 1) Patients who are unable to understand the RM procedure or do not have relatives who can take care of it and, 2) Patients who reject RM and prefer to attend consultation.

Criteria for participation in the study

a) **Inclusion criteria:** 1) Be 18 years of age, 2) Have a PM implanted in any of the two types of monitoring: RM versus HM and 3) Understand and be able to properly perform self-monitoring at home.
b) **Exclusion criteria**: 1) Be participating in another study and / or 2) Refuse to participate in the study.

Environment

The choice of hospitals - *Hospital de Poniente (Spain) / Nordland Hospital (Norway)* - is due to the number of implanted pacemakers (80-90PM/year), but they also differ in other respects such as the reference population *Hospital de Poniente (256,000 inhabitants) vs Nordland Hospital (180,000 inhabitants),* lifestyles, North-South relationship, different health systems, etc.).

These issues and others have motivated us to perform a comparative study of the same technology (remote pacemaker monitoring), applied in two countries at very different priori.

Selected variables from the medical history records.

For pacemakers, the selected parameters are indicative of the proper functioning of the cardiac device:

1. Battery impedance.
2. Battery Voltage.
3. Atrial electrode impedance.
4. Ventricular electrode impedance.
5. Atrial pacing threshold.
6. Ventricular stimulation threshold.
7. Voltage of the intrinsic atrial signal.
8. Voltage of the intrinsic ventricular signal.
9. Histogram of heart rates.
10. Percentage of the intrinsic activity with regard to the stimulated atrium.
11. Percentage of the intrinsic activity with regard to the stimulated ventricle.
12. Presence of arrhythmias since the last check-up.
13. Will a direct examination be necessary?

Socio-demographic variables, will allow us to perform a statistical analysis of the following social characteristics of patients with pacemakers:

1. Gender.
2. Date of birth / age.
3. City / Town.
4. Consumption of tabacco.
5. Alcohol intake.

Clinical variables, will allow us to establish the following clinical characteristics (medical history, background, hospitalisation, examinations underwent etc.) shown by patients throughout the process:

1. Main diagnosis.
2. Duration of the pathology.
3. Other pathologies.
4. Type of pharmacological treatment they receive.
5. Number of hospitalisations (in-patient treatments) in the previous year.
6. Number of hospitalisation days (in-patient treatments) in the previous year.
7. The unit that has referred the patient for a pacemaker consultation.
8. Date of the pacemaker implantation.
9. Number of hospitalisation days after the pacemaker implantation.
10. Type of the pacemaker implanted.
11. Blood pressure/ weight / temperature / diabetes.
12. Number out-patient examinations.
13. Number of cardiac events.

Regardless of the type of monitoring, the parameters to be measured shall be the same. Access to these variables will allow us to obtain the pacemaker values at different times, which, in turn, will let us establish if there are any differences between the two groups.

In the identification of the *health-related quality of life (HRQoL)* , two questionnaires will be administered:

1. *Generic questionnaire, EuroQol-5D (EQ-5D)*[29-33] (Annex 1):

   - ✓ It is a tool that creates an index value to be used in studies of cost-effectiveness, especially in the allocation of health resources[31].

   - ✓ The following dimensions of health are measured: mobility, self-care, usual activities, pain/discomfort and anxiety/depression.

2. *Specific questionnaire, Duke Activity Index* [33-36] (Annex 2):

- ✓ It is an instrument that assesses the functional capacity of patients with cardiovascular disease.
- ✓ It has the ability to predict the quality of life of patients with cardiac disease.
- ✓ The following dimensions were measured: functional capacity in domestic, industrial, sexual and recreational or leisure activities.

In both cases:

- ✓ They will be administered the day before implantation and at months 1 and 6 after surgery.
- ✓ The collection of information is done through personalised interviews during hospitalisation, patient attendance to medical examinations and/or telephone interviews.
- ✓ The questionnaires have "closed" questions. The same questionnaire regardless of the way in which the information is collected (personal or telephone interview) will be used.
- ✓ They are internationally validated questionnaires[30-36].
- ✓ They are tools commonly used in the Economic Evaluation of Health Technologies[30-35, 37,38].

Ethical aspects of the research

It meets the precepts of ethics and research set by the Helsinki Declaration of the World Medical Association. In addition, personal data obtained will be confidential and shall be treated in accordance with that established in the laws on the protection of personal data, both in Norway and Spain. The database obtained from this study will be kept for 3 years in order to publicise the results of the research to the scientific community. After this period the database will be destroyed.

Attached:

1) Informational letter (Annex 3) and Adult consent (Annex 4).

Primary Results

Incidences of complications within the first 6 months after implantation will be assessed. Different events such as complications related to heart function, surgery (problems of stimulation/ detection), arrhythmias, muscle stimulation, and death are included.

Type, cause, duration and response to the cardiac event, monitoring visits. All medical interventions and medication changes related to the occurrence of events will also be collected.

Secondary outcomes

In this section the results of the quality of life perceived by patients with PMs in 3 different times (pre-implant and at 1 and 6 months post-implant) will be collected. And both formal and informal costs, incurred in the same period. This will allow us to compare the cost-effective relationship between HM and RM.

The perceived quality of life was measured using the generic questionnaire EuroQol-5D (EQ-5D) and the specific questionnaire Duke Activity Index. In this we can access the changing perception of health.

Data Analysis

Analysis of the sociodemographic characteristics and population clinics

From the data collected in the list of the patient's medical history can be obtained. Patient sociodemographic data (sex, year of birth, place of residence, etc.), clinical variables (number and type of cardiac event, tobacco, DM,

alcohol, weight, other pathologies, drugs, etc.) and the variables / parameters to monitor, grouped into two groups (dependent and independent variables), will be included. From the characteristics of each of these records and, as an average weighted by the number of individuals comprising it.

The mean ± standard deviation and range will be calculated to describe continuous variables and proportions for quantitative variables. The differences between both types of cardiac monitoring, from the $\chi 2$ test for categorical variables will be compared. Parametric and nonparametric tests will be carried out for continuous variables depending on the normality assumption. In cases in which the assumption of normality cannot be made, the nonparametric Mann-Whitney test for continuous variables will be performed.

EuroQol-5D questionnaire (EQ-5D) and HRQoL Duke Activity Index

HRQoL scores between subgroups of clinical interest (age, sex, functional class, etc.) with proof of Student's t or variance analysis EuroQol-5D were compared, according to the variable being analyzed for components summaries (EQ-5D) and through Mann-Whitney or Kruskal-Wallis nonparametric tests for the Duke Activity Index scores. The confidence level is set at 95%.

A linear regression to evaluate the quality of previous life will be carried out, the final values and, evaluate the differences.

Limitations

In relation to the period of data collection, we must consider the risk that once this period is over, the two groups will not be comparable. To overcome this situation, it is proposed to increase the period of data collection and therefore the study period.

The absence of randomisation makes it difficult to establish causal effects and the degree of internal validity is reduced while increasing the potential for generalisation of the results[39].

General objective 2: To analyse the costs of RM versus HM, of PM cardiac patients.

Methodology

Qualitative and quantitative design, in which the following tools are used:

In-depth interviews of those responsible for the Cardiology and Accounting Units, of both hospitals with the aim of identifying medical costs both direct and indirect, which are grouped according to their origin.

Table 2. Direct Cost vs indirect cost

Direct Costs	Indirect Costs
Consultation costs (area, electricity, furniture, sanitary fungible investments …)	Displacement (means of transport, km from home to the hospital).
Personnel costs (doctors, nurses, administrative …)	Accommodation, meals …
PM costs	Costs per day lost.
Pharmaceutical costs (cardiovascular drug consumption)	
Cost per day of hospital stay	
Time spent on each patient	

The **identification of the costs associated with informal care** will be obtained from the Caregiver Questionnaire belonging to Survey on Disability, Personal Autonomy and Dependency (DPAD) of the National Statistics Institute[40] from Spain adapted for Norway.

DPAD questionnaire allows us to find out: 1) demographic and social characteristics of caregivers, 2) Level of professionalism, 3) Time spent and types of care, 4) Difficulties in providing care 5) Health status and, 6) Professional aspects, family or leisure of those who have had to do without due to engaging in caregiving.

DPAD tool allows the collection of information may be made by personal interview and / or phone to the primary caregivers if any (See Annex 5)[41.42] and will be administered 1 month after implantation of the PM, coinciding with the period in which most of the implanted patients needed the help of a caregiver.

For the collection and evaluation of the corresponding informal costs to the rest of the study period, we will use the following tools: the clinical history, patient interviews and information provided by the accounting unit.

The costs are estimated in terms of real prices of 01/01/2015, taking into account the perspective of the National Health System and from the perspective of society (informal). Consumption of medicines and resources used/patient (including monitoring visits to the hospital, interventions and hospitalisations related to the implant) will be collected.

The result of multiplying the average resource usage with actual prices may be estimated costs / patient.

Data Analysis

To determine the utilisation and unit value of direct costs and indirect costs associated with informal caregivers, the

data provided by the following will be used: 1) Ministry of Health, Social Services and Equality (Spain) and Ministry of Health and Care Services (Norway), 2) Official Gazettes, 3) Department of Accountings at both hospitals and 4) National Statistical Institutes of Spain and Norway, up to 1 September 2014.

In estimating the costs of informal care, the time they spend in care is taken into consideration. The ***replacement cost*** method will be used, which consists in the measuring the costs that the changing of informal carers for professionals would entail. The average cost per hour of home help service in both countries will be taken into account in the assessment of the care. The costs are measured in Euros (€) and effectiveness will be measured in years of quality-adjusted life year (QALY). This index is usually obtained through the generic EuroQol-5D questionnaire (EQ-5D), which is an instrument that is internationally endorsed[2.3].

General objective 3: To assess the impact and cost effectiveness in the medium and long-term of RM vs HM of PMs: Markov's mathematical model.

Methodology

A ***Decision Tree*** will be built which through graphical representations in diagrams will help us show, in a modeled and clear way, all the alternatives, events and results of applying each type of monitoring (remote vs hospital).

Basic aspects to consider in the construction of a decision tree:

1) Type of monitoring (remote vs hospital).
2) Effectiveness (perceived quality of life and satisfaction)
3) Costs (health and informal)
4) Time horizon, divided into study cycles (annual).
5) Software: MS Excel

We have chosen the Markov model mathematical simulation over other instruments because it is one of the main tools in health economics to estimate the costs and developments in the medium and long term of chronic diseases[43.44].

An analytical model of decision shall be taken both from the health and social perspective that helps us to estimate the evolution in the medium and long term, to provide greater information on the benefits of implementing an intervention and associated costs thereto. Analytical models help decision makers (health, political and social) to systematise the various expected and controllable alternatives. Furthermore, in cases where we are in uncertain situations, they will help us choose the option that maximizes the expected results.

Data Analysis

To analyze the results of the Markov processes, we will use ***simulation through cohort***. The main reason is a very intuitive simulation in which individuals belonging to each cohort must pass over time to other states, being in most cases and less healthy, ending with the death of the subject. The expected outcome in terms of median survival time or stay in a certain state, is estimated by dividing the number of cycles added by the entire cohort between the size thereof [8.40].

To the ***sensitivity analysis, globally accepted common sense and guides or checklists*** will be applied, to remove or correct uncertainties that may arise. Keep in mind that in the evaluation of health technologies, ***the causes of uncertainty*** may be due to the variability of the available data, generalisation of the results, and extrapolation of events, and use of inappropriate analytical methods[8,40,41].

References

1. Asociación de Economía de la Salud. Capítulo 1. El impacto en la salud de la población de la crisis económica y las políticas para abordarla. En "Sistema Nacional de Salud: diagnóstico y propuestas de avance". 2013 (citado 25 Ene 2014). Disponible en: http://www.sespas.es/adminweb/uploads/docs/V09_Crisis_economica_y_salud_SESPAS%20_3_X.pdf

3. García-Altés A, Navas E, Soriano M. Evaluación económica de intervenciones de salud pública. Gacet Sanit. 2011;25(Supl 1):S25-31.

3. Fundación Gaspar Casal, para la investigación y el desarrollo de la salud. Gestión Clínica. Madrid (España): Fundación Gaspar Casal; 2011.

4. Gold M, Siegel J, Russell L, Weinstein M. Cost – effectiveness in health and medicine. Oxford (England): Oxford University Press. 1996

5. Drummond MF, O'Brien BJ, Stoddart GL, Torrance GW. Methods for the Economic Evaluation of Health Care Programs. Oxford University Press, 1997. 385 p

6. López-Bastida J. Economía de la salud: el coste de la enfermedad y la evaluación económica en las enfermedades respiratorias. 2006. Arch Bronconeumol.42(5):207-10.

7. Tarride J, Morgan L, DesMeules M, Luo W, Burke N, O'Reilly D, et al. A review of the cost of cardiovascular disease. Can J Cardiol. 2009;25(6):195–202. Publicación electrónica Jun 2009.

8. European Heart Network (Internet). European Heart Network AISBL. 2008 (citado 23 Nov 2013). Disponible en: http://www.ehnheart.org/cvd-statistics.html

8. García F, Porres, JM, Martín-Chico F. Dispositivos eléctricos y monitorización remota. Rev Urug Cardiol. 2013:28(1): 63-70

9. Medtronic Ibérica S.A. (s.f.). Más que corazón. 2011 (citado 11 Oct 2013). Disponible en: http://masquecorazon.com/monitorizaciondistancia.html

11. Elsner C, Sommer P, Piorkowski C, Taborsky M, Neuser H, Bytesnik J. A prospective multicenter comparison trial of Home Monitoring against regular follow-up in Madit II patients: additional visits and cost impact. Comput Cardiol. 2006;33:241-4.

12. Osca J, Sancho M, Navarro J, Cano O, Raso R, Castro J, et al. Fiabilidad técnica y seguridad clínica de un sistema de monitorización remota de dispositivos cardiacos antiarrítmicos. Rev Esp Cardiol. 2009;62(08):886-95.

13. De Cock C, Elders J, van Hemel M, van den Broek K, van Erven L, de Mol B, et al. Remote monitoring and follow-up of cardiovascular implantable electronic devices in the Netherlands. An expert consensus report of the Netherlands Society of Cardiology. Neth Heart J. 2012;20(2):53–65.

14. Folino A, Breda R, Calzavara P, Migliore F, Iliceto S, Bruja G. In-home controls of pacemakers in debilitated elderly patients. Geriatr Gerontol Int. 2012;12:30-5.

15. Folino A, Breda R, Calzavara P, Borghetti F, Comisso J, Iliceto S, et al. Remote follow-up of pacemakers in a selected population of debilitated elderly patients. Europace. 2013;15:382-7.

16. Raatikainen M, Uusimaa P, van Ginneken M, Janssen J, Linnaluoto M. Remote monitoring of implantable cardioverter defibrillator patients: a safe, time-saving, and cost-effective means for follow-up. Europace. 2008;10(10):1145-51.

17. Fauchier L, Sadoul N, Kouakam C, Briand F, Chauvin M, Babuty D, et al. Potential cost savings by telemedicine-assited long-term care of implantable cardioverter difibrillator recipients. Pacing Clin. Electrophysiol. 2005;28(Suppl.1):S255-9.

18. Al-Khatib S, Piccini J, Knight D, Stewart M, Clapp-Channing N, Sanders G. Remote monitoring of implantable cardioverter defibrillators versus quarterly device interrogations in clinic: results from a ramdomized pilot clinical trial. J. Cardiovasc. Electrophysiol. 2010;21(5):545-50.

19. Linde C, Abraham W, Gold M, St John Sutton M, Ghio S, Daubert C. Ramdomized trial of cardiac resynchronization in mildly symptomatic heart failure patients and in asymptomatic patients with left ventricular dysfuction and previous heart failure symptoms. J Am Coll Cardiol. 2008;52:1834-43.

20. Moss A, Hall W, Cannom D, Klein H, Brown M, Daubert J, et al. Cardiac-resynchronization therapy for the prevention of heart-failure events. N Engl J Med. 2009;361:1329-38.

21. Coma-Samartín R, Ruiz-Mateas F, Fidalgo-Andrés M, Leal del Ojo-González J, Pérez-Álvarez. Registro Español de Marcapasos. X Informe Oficial de la Sección de Estimulación Cardiaca de la Sociedad Española de Cardiología (2012)). Rev Esp Cardiol. 2013;66:959-72..

22. US Congress, Office of Technology Assessment. Assessing the Efficacy and Safety of Medical Technologies. Washington DC (USA): US Government Printing Office; 1978

23. Lanuza A. Plataforma Española de Innovación en Tecnología Sanitaria. Barcelona: federación española de empresas de tecnología sanitaria; 2010 (citado 26Nov 2013). Disponible en: http://medicamentos-innovadores.org/sites/default/files/medinnovadores/Espa%C3%B1ol/Eventos/2010/23-24-02-10/Angel%20Lanuza.pdf

24. Oliva J, Del Llano J, Sacristán J. La evaluación económica de tecnologías sanitarias en España: Situación actual y utilidad como guía en la asignación de recursos sanitarios. ICE Economía de la Salud. 2003; 804:155-167.

25. MedlinePlus. Marcapasos cardíaco; 2010 (citado 26 Nov 2013). Disponible en: http://www.nlm.nih.gov/medlineplus/spanish/ency/article/007369.htm

26. Haug B, Kjelsberg K, Lappegård KT. Pacemaker implantation in small hospitals: complication rates comparable to larger centres. Europace. 2011;13:1580–6.

27. Kamphuis H, Leeuw J, Derksen R, Hauer R, Winnubst J. Implantable cardioverter defibrillator recipients: quality of life in recipients with and without ICD shock delivery: a prospective study. Europace. 2003;5(4):381–9.

28. Broek K, Denollet J, Nyklicek I, Voort P. Psychological reaction to potential malfunctioning of implantable defibrillators. PacingClinElectrophysiol. 2006;29(9):953–6.

29. Gènova-Maleras R, Álvarez-Martín E, Catalá-López F, Fernández de Larrea-Baz N, Morant-Ginestar C. Aproximación a la carga de enfermedad de las personas mayores en España. Gac. Sanit. 2011;25 Supl:S47-50.

30. European Heart Network (Internet). European Heart Network AISBL. 2008 (citado 23 May 2012). Disponible en: http://www.ehnheart.org/cvd-statistics.html

31. EuroQol Group. EuroQol . A new facility for the measurement of health-related quality of life. Health Policy. 1990;16:199-208.

32. Enden T, Wik HS, Kvam AK, Haig Y, Kløe NE, Sandsed PM. Health-related quality of life after catheter-directed thrombolisys for deep vein thrombosis: secundary outcomes of the randomised, non-blinded, parallel-group CaVenT study. BMJ Open. 2013;3(8):e002984. doi: 10.1136/bmjopen-2013-002984.

33. Badia X, Roset M, Montserrat S, Herdman M, Segura A. La versión española del EuroQol: descripción y aplicaciones. Medicina Clínica. 1999;112(Supl 1):S79-86.

34. Fan X, Lee KS, Frazier SK, Lennie TA, Moser DK. Psychometric testing of the Duke Activity Status Index in patients with heart failure. Eur J Cardiovasc Nurs; 2014. doi: 10.1177/1474515114523354

35. Hlatky MA, Boineau RE, Higginbotham MB, Lee KL, Mark DB, Califf RM, et al. A brief self-administered questionnaire to determine functional capacity (the Duke Activity Status Index). Am J Cardiol. 1989;64:651-4.

36. Alonso J, Permanyer-Miralda G, Cascant P, Brotons C, Prieto L, Soler-Soler J. Measuring functional status of

chronic coronary patients. EuropeanHeartJournal. 1997;18:414-9.

37. Permanyer C, Brotons C, Ribera A, Alonso J, Cascant P, Moral I. Resultados después de cirugía coronaria: determinantes de calidad de vida relacionada con la salud postoperatoria. RevEspCardiol. 2001;54:607-16.

38. Callejo D, Martín C, Guerra M, Blasco JA. Terapia de resincronización cardiaca: Evaluación económica. Madrid: Unidad de Evaluación de Tecnologías Sanitarias, Agencia Laín Entralgo; 2010.

39. 3datos. Los diseños de investigación en las ciencias de la salud. 2011 (citado 17 May 2013). Disponible en: http://3datos.es/wp-content/uploads/2011/10/MET-2-Dise%C3%B1os.pdf

40. Instituto Nacional de Estadística. Salud. 2012 (citado 01 Ago 2012). Disponible en: http://www.ine.es/jaxi/menu.do?type=pcaxis&path=/t15/p418&file=inebase&L=0

41. Oliva J, Osuna R. Los costes de los cuidados informales en España. Presupuesto y Gasto Público. 2009;56:163-181.

42. Instituto Nacional de Estadística. Encuesta de Discapacidad, Autonomía personal y situaciones de Dependencia (EDAD). Metodología. 2010 (citado 15 Jul 2012). Disponible en: http://www.ine.es/metodologia/t15/t1530418.pdf

43. Bala M, Mauskopf J. Optimal Assignment of Treatments to Health State Using a Markov Decision Model: An Introduction to Basic Concepts. Pharmacoeconomics. 2006;24(4):345-354.

44. Rubio-Terrés C, Echevarría A. Modelos de Markov: Una herramienta útil para el análisis farmacoeconómico. Pharmacoeconomics. 2006;3(Supl 2):S71-8.

Have the involved groups and researchers cooperated before? If yes, what shall this project provide to the existing relation?

As mentioned above, the project has been developed in Spain since 2012. All Spanish institutions have collaborated since the implementation of the project.

However, this project presented in the call NILS Mobility Projects arising from the introduction of the partner of the University of Tromsø to expand the project to another country like Norway. So this project will facilitate the internationalisation of the research and the possible comparative analysis between Spain and Norway.

Shall this project be the basis for establishing a new cooperation?

Yes, this project will be the basis for establishing new collaborations between groups in Norway and Spain. The components of the various teams of researchers from Spain and Norway share the interest and professional practice in various fields such as cardiology, quality of life, health communication, costs, new health technologies, mathematics and of course, improving the welfare and quality of life of people.

6. CHRONOGRAM / CRONOGRAMA

AIM	MONTHS												
	1	2	3	4	5	6	7	8	9	10	11	12	13
1													
2													
3													

	Meetings in Nordland Hospital (Bodø) with the Norwegian team investigation and resolution of administrative issues (University of Tronsø).
	Collection, monitoring and effectiveness analysis.
	Identification, collection and costs analysis.
	Economic Impact Analysis: Markov mathematical model.
	Termination administrative issues with the University of Tronsø and Bodø Nordland Hospital.

7. RISK ASSESSMENT AND MITIGATION PLAN

Identify and assess the relevant risks that may affect the achievement of expected results and/or the development of the planned activities. For each risk identified, describe it, assess the probability of the risk to happen and the impact it would have, and describe how you plan to mitigate it.

Aim /Expected result	Description of the risk	Probability (high/ medium/low)	Impact (high/medium/low)	Mitigation plan *Plan de mitigación*
Analyse clinical and sociodemographic characteristics. / Know if there are differences between both groups.	No have access to the clinical histories of patients.	low	medium	Ask for the permission corresponding to the regional ethics committee from Norway.
Assess perceived quality of life. / Know if there are differences between the two types of tracking.	After completing the data collection period, to check that the groups are not comparable.	low	medium	Increase the data collection period.
Identify and assess healthcare costs from both modalities. / It show us whether there are significant differences between the groups.	Not access the accounting department of the hospital.	low	medium	Ask for corresponding permit in the accounting department of the Nordland Hospital.
Analyze the informal costs of both types of monitoring. / Know if there are differences between both groups.	Not participation of patients and carers.	low	low	Explain to patients and caregivers, how important their participation in the study.

8. COMMUNICATION PLAN

The results of each of the three general objectives will be presented the following way:

1) Manuscripts will be published in scientific journals indexed in JCR:

2) 1 Clinical Practice Guideline, chapters and 1 book that collect the results of each of the objectives included in the study.

3) Oral Communications & posters will be presented in different International Congresses on Cardiology, Health economics, Telemedicine and Geriatrics:

Although the dissemination plan seems ambitious, no ceases to be realistic given that most of the researchers who are on the team have publications in JCR journals.

Spørreskjema om helse

Norsk versjon, for Norge

(Norwegian version for Norway)

Supported by a grant from Iceland, Liechtenstein and Norway through the EEA Financial Mechanism.
Operated by Universidad Complutense de Madrid.
NILS management service +34 91 394 4385 e-mail: nilss@rect.ucm.es

Vis hvilke utsagn som passer best på din helsetilstand i dag ved å sette et kryss i en av rutene utenfor hver av gruppene nedenfor.

Gange

Jeg har ingen problemer med å gå omkring. ❑

Jeg har litt problemer med å gå omkring. ❑

Jeg er sengeliggende. ❑

Personlig stell

Jeg har ingen problemer med personlig stell. ❑

Jeg har litt problemer med å vaske meg eller kle meg. ❑

Jeg er ute av stand til å vaske meg eller kle meg. ❑

Vanlige gjøremål *(f.eks. arbeid, studier, husarbeid, familie- eller fritidsaktiviteter).*

Jeg har ingen problemer med å utføre mine vanlige gjøremål ❑

Jeg har litt problemer med å utføre mine vanlige gjøremål. ❑

Jeg er ute av stand til å utføre mine vanlige gjøremål. ❑

Smerte/ubehag

Jeg har verken smerte eller ubehag. ❑

Jeg har moderat smerte eller ubehag. ❑

Jeg har sterk smerte eller ubehag. ❑

Angst/depresjon

Jeg er verken engstelig eller deprimert. ❑

Jeg er noe engstelig eller deprimert. ❑

Jeg er svært engstelig eller deprimert. ❑

Best tenkelige
helsetilstand

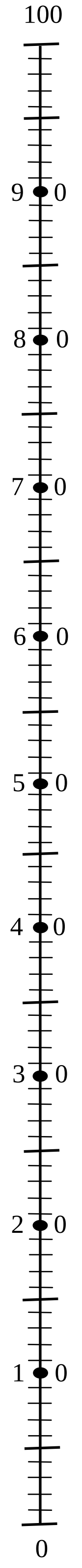

Verst tenkelige
helsetilstand

For å hjelpe folk til å si hvor god eller dårlig en helsetilstand er, har vi laget en skala (omtrent som et termometer) hvor den beste tilstanden du kan tenke deg er merket 100 og den verste tilstanden du kan tenke deg er merket 0.

Vi vil gjerne at du viser på denne skalaen hvor god eller dårlig helsetilstanden din er i dag, etter din oppfatning. Vær vennlig å gjøre dette ved å trekke en linje fra boksen nedenfor til det punktet på skalaen som viser hvor god eller dårlig din helsetilstand er i dag.

Din egen
helsetilstand
i dag

Supported by a grant from Iceland, Liechtenstein and Norway through the EEA Financial Mechanism.
Operated by Universidad Complutense de Madrid.
NILS management service +34 91 394 4385 e-mail: nilss@rect.ucm.es
