# Supplementary material for: Cost–utility analysis of telemonitoring versus conventional hospital-based follow-up of patients with pacemakers. The NORDLAND randomized clinical trial
Source: PLoS One. 2020 Jan 29;15(1):e0226188. doi: 10.1371/journal.pone.0226188 (PMC6988929; doi:10.1371/journal.pone.0226188)
Supplement: S2 Appendix — (PDF) [file pone.0226188.s002.pdf]

Cost-utility analysis of telemonitoring versus conventional hospital-based follow-up of patients with pacemakers. The NORDLAND randomized clinical trial.

Lopez-Villegas A, Catalan-Matamoros D, Peiro S, Tore Lappegard K, Lopez-Liria R.

DATA COLLECTION FORMS (ENGLISH VERSION)

DATA COLLECTION CLINICAL HISTORY

Patient Number **Routine / Ordinary**

BEFORE IMPLANT				
Symptoms	Syncope	Dizziness	Dyspnea	
Origin	Emergency wards	Cardiology outpatient clinic	Primay healthcare	Other hospital departments
Indication for pacemakers implant	Sinus node dysfunction	Atrioventricular block	Chronic AF with Bradycardia	Other

AFTER IMPLANT				
Stimulation	DDDR		VVIR	
	**6 months**		**12 months**	
Total number of hospital transmissions				
Total number of transmissions from home	Routine	Extra	Routine	Extra
Cardiovascular events:				
1. Total number of calls/letters sent to the patient				
2. Total number of changes of medication				
3. Total number of changes of reprogramming				
Hospitalization after implant				
Total number of hospitalization days				
Reasons for the hospitalization				

DATA COLLECTION CLINICAL HISTORY

Patient Number	Routine / Ordinary	

Comorbidities (at the time of implant)		
Hypertension	Yes	No
Diabetes mellitus	Yes	No
Tachyarrhythmia	Yes	No
Dislipidemia (according to use of medicines)	Yes	No
Obesity (BMI > 30)	Yes	No
Others:		
**Medical treatment (at the time of implant)**		
Antiaggregants	Yes	No
Anticoagulants	Yes	No
Antiarrhythmic	Yes	No
Antihypertensives	Yes	No

By placing a tick in one box in each group below, please indicate which statements best describe own health state today

Mobility

I have no problems in walking about ❑

I have some problems in walking about ❑

I am confined to bed ❑

Self-Care

I have no problems with self-care ❑

I have some problems washing or dressing myself ❑

I am unable to wash or dress myself ❑

Usual activities *(e.g. work, study, homework, family or leisure activities).*

I have no problems with performing my usual activities ❑

I have some problems with performing my usual activities ❑

I am unable to perform my usual activities ❑

Pain/Discomfort

I have no pain or discomfort ❑

I have moderate pain or discomfort ❑

I have extreme pain or discomfort ❑

Anxiety/Depression

I am not anxious or depressed ❑

I am moderately anxious or depressed ❑

I am extremely anxious or depressed ❑

To help people say how good or bad health state is, we have drawn a scale (rather like thermometer) on which the best state you can imagine is marked 100 and the worst state you can imagine is marked 0.

We would like you to indicate on this scale how good or bad your own health is today, in your opinion. Please do this by drawing a line from the bow below to whichever point on the scale indicates how good or bad your health state is today.

Your own health state today

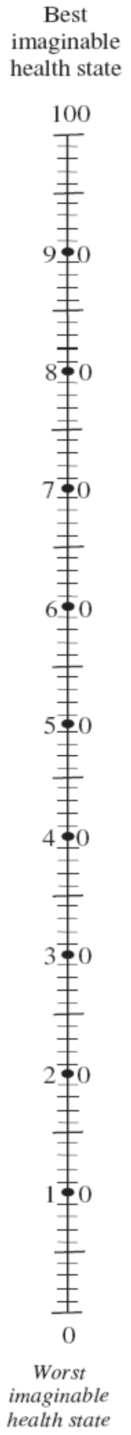

Survey on telehealth patient experience

(adapted version from Abrams &Geier, 2006; Bas-Villalobos, 2010)

Both types of participants: hospital and home-monitored

1. How many kilometres is your home from hospital?

2. How much time does it take youto attend a cardiology consultation? (number of Hours since leaving your home for going to hospital, until you arrive back home, including 2-ways travel, waiting and consultation times)

   □ < 1 hour □ 1 - 2 hours □ 2 - 3 hours □ 3 – 4 hours □ > 4 hours

3. What type of transport do you use to travel to hospital?

   □ Public transport □ Own car □ Ambulance □ Taxi □ Other: ___________

4. Which is your labour situation now?

   □ Working □ Unemployed □ Pensionist □ Sickleave □ Other: __________

5. Do you need to be accompanied by any relative or friend to attend the cardiology consultation at hospital?

   □ No □ Yes

6. Which is the labour situation of your accompanying person?

   □ Working □ Unemployed □ Pensionist □ Sickleave □ Other: __________

7. Has you or the accompanying person any expenses when travelling to hospital?

   □ No □ Yes (approx. NOK____________)

8. How many times have you phoned to the pacemakers office at hospital?

   □ None □ 1 □ 2 □ More than 2

9. How many times have you atended the emergency ward for a problema related to your pacemaker in either the hospital or primary healthcare centre?

   □ None □ 1 □ 2 □ More than 2
