# Supplementary material for: Cost–utility analysis of telemonitoring versus conventional hospital-based follow-up of patients with pacemakers. The NORDLAND randomized clinical trial
Source: PLoS One. 2020 Jan 29;15(1):e0226188. doi: 10.1371/journal.pone.0226188 (PMC6988929; doi:10.1371/journal.pone.0226188)
Supplement: S3 Appendix — (PDF) [file pone.0226188.s003.pdf]

Cost-utility analysis of telemonitoring versus conventional hospital-based follow-up of patients with pacemakers. The NORDLAND randomized clinical trial.

Lopez-Villegas A, Catalan-Matamoros D, Peiro S, Tore Lappegard K, Lopez-Liria R.

DO FILE (STATA)

**** PATIENTS' CLINICAL CHARACTERISTICS AT BASELINE (TABLE 1)***
***** "summarize" command (for describing) and "ttest" command (for differences between groups) for quantitative variables, were used, and "tab" command with chi square or Fisher's exact test for qualitative variables.***

***** Age and sex.***
```
by ARM_N, sort: summarize AGE, detail
ttest AGE, by(ARM_N)
tab SEX ARM_N, row col chi exact
prtest SEX, by(ARM_N)
```

***** Pacing indication, symptoms and stimulation.***
```
tab ORIGIN ARM_N, row col chi exact
tab INDICATION ARM_N, row col chi exact
tab SYMPTOMS ARM_N, row col chi exact
tab STIMULATION ARM_N, row col chi exact
```

***** Comorbidities: high blood pressure, coronary heart disease, diabetes, tachyarrhythmia, obesity (BMI>30), other, none.***
```
tab C_HBP ARM_N, row col chi exact
tab C_CHD ARM_N, row col chi exact
tab C_TACHYARRHYT ARM_N, row col chi exact
tab C_DISLYP ARM_N, row col chi exact
tab C_DM ARM_N, row col chi exact
tab C_OBESI ARM_N, row col chi exact
tab C_OTHER2 ARM_N, row col chi exact
tab C_NONE ARM_N, row col chi exact
```

***** Treatment (basal): antiplatelet agents, oral anticoagulants, anti-arrhythmic drugs, anti-hypertensive drugs.***
```
tab TR_PLAQ ARM_N, row col chi exact
tab TR_OAC ARM_N, row col chi exact
tab TR_ARRIT ARM_N, row col chi exact
tab TR_ANTIHT ARM_N, row col chi exact
```

***** EQ5D basal (month 0) and at first month.***
```
by ARM_N, sort: summarize EQ5D_MONTH_0, detail
ttest EQ5D_MONTH_0, by(ARM_N)
by ARM_N, sort: summarize EQ5D_MONTH_1, detail
ttest EQ5D_MONTH_1, by(ARM_N)
```

```
* COST INPUTS, COSTS PER PATIENT YEAR, AND QUALITY OF LIFE OUTCOMES (TABLE 2)
** "ttest" command for differences between groups was used.

** UTILIZATION
** In-office visits, patient/year
ttest n_HOSP_TRANSM, by(ARM_N)
** Pacemaker transmission, patient/year
ttest t_TRANSM, by(ARM_N)
** Physician time, min/patient
ttest PHYS_MIN, by(ARM_N)
** Hospitalization days
ttest HOSP_DAYS, by(ARM_N)
** Distance home/hosp (Km)
ttest DISTANCE, by(ARM_N)
** Patients' time (travel & visits)
ttest PATIENT_TIME, by(ARM_N)

** NHS COSTS
** Physician costs
ttest COST_DR, by(ARM_N)
** Consultation room costs
ttest COST_ROOM, by(ARM_N)
** Hospitalization costs
ttest COST_HOSP, by(ARM_N)
** Ambulance transport costs
ttest COST_AMBUL, by(ARM_N)
** Total NHS costs
generate tCOSTS_NHS = COST_DR + COST_ROOM + COST_HOSP + COST_AMBUL
label variable tCOSTS_NHS "Total costs NHS perspective"
format %9.2f tCOSTS_NHS
ttest tCOSTS_NHS, by(ARM_N)

** PATIENT COSTS
** Patient travel costs
ttest COST_P_TRAVEL, by(ARM_N)
** Patient travel+waiting costs
ttest COST_P_TRAV_WAIT, by(ARM_N)
** Other transport costs
ttest COST_P_OTH_TRAV, by(ARM_N)
** Accompanying person costs
ttest COST_P_ACOMP, by(ARM_N)
ttest HOSP_CONS, by(ARM_N)
** Total patient costs
generate tCOST_PATIENT = COST_P_TRAV_WAIT + COST_P_OTH_TRAV + COST_P_ACOMP
label variable tCOST_PATIENT "Total patient&family costs"
```

```
format %9.2f tCOST_PATIENT
ttest tCOST_PATIENT, by(ARM_N)
```

***** TOTAL (NHS + PATIENT&FAMILY) COSTS***

```
generate tCOSTS = tCOSTS_NHS + tCOST_PATIENT
label variable tCOSTS "Total costs (NHS+Patients&family)"
ttest tCOSTS, by(ARM_N)
```

***** OUTCOMES***

***** Hospitalizations and deaths***

```
by ARM_N, sort: ci HOSPITALIZ, binomial total
prtest HOSPITALIZ, by(ARM_N)
by ARM_N, sort: ci DEATH, binomial total
prtest DEATH, by(ARM_N)
```

***** Quality of life at 6 and 12 months***

```
ttest EQ5D_MONTH_6, by(ARM_N)
by ARM_N, sort: ci HOSPITALIZ, binomial total
ttest EQ5D_MONTH_12, by(ARM_N)
```

***** Generating and analyzing QALYs***

***** Generating QALY1 as lineal interpolation between EQ5D weights at month 1 and 6, between month 6 and 12, and between the two resultant values.***

```
generate QALY_1 = (((EQ5D_MONTH_1 + EQ5D_MONTH_6)/2) + ((EQ5D_MONTH_6 +
EQ5D_MONTH_12)/2))/2
label variable QALY_1 "QALYs"
format %9.4f QALY_1
ttest QALY_1, by(ARM_N)
```

**** COSTS PER PATIENT/YEAR AND COST-UTILITY ANALYSIS (TABLE 3)***

***** Generating cost per QALY variables and testing differences.***

```
generate NHSCOST_Q1 = tCOSTS_NHS/QALY_1
replace NHSCOST_Q1=0 if NHSCOST_Q1==.
generate TCOST_Q1 = tCOSTS/QALY_1
replace TCOST_Q1=0 if TCOST_Q1==.
label variable NHSCOST_Q1 "Cost/QALY NHS perspective"
format %9.2f NHSCOST_Q1
label variable TCOST_Q1 "Total Cost/QALY"
format %9.2f TCOST_Q1
ttest NHSCOST_Q1, by(ARM_N)
ttest TCOST_Q1, by(ARM_N)
```

***** Cost-utility analysis (total NHS costs and total costs) using heabs stata command (with a WTP threshold of 30000 and 50000 euros).***

```
heabs tCOSTS_NHS QALY_1, res(bene) int(ARM_N) w2p (30000)
heabs tCOSTS_NHS QALY_1, res(bene) int(ARM_N) w2p (50000)
heabs tCOSTS QALY_1, res(bene) int(ARM_N) w2p (30000)
```

```
heabs tCOSTS QALY_1, res(bene) int(ARM_N) w2p (50000)
```

**** The probabilistic analysis was carried out with the HDS online calculator (http://www.healthstrategy.com/evpiinb/evpiinb01.htm)***

**** SUPPLEMENTAL ANALYSIS (TABLE S2.2. COSTS PER PATIENT-YEAR EXCLUDING HOSPITALIZATION DAYS COSTS)***

```
generate tCOST_NHS_W = tCOSTS_NHS - COST_HOSP
generate tCOST_W = tCOSTS - COST_HOSP
label variable tCOST_NHS_W "Total costs NHS perspective W/O hospitalization costs"
label variable tCOST_W "Total costs W/O hospitalization costs"
format %9.2f tCOST_NHS_W
format %9.2f tCOST_W
ttest tCOST_NHS_W, by(ARM_N)
ttest tCOST_W, by(ARM_N)
```

***** Generating cost (w/o hospitalization days) per QALY variables.***

```
generate NHSCOSTW_Q1 = tCOST_NHS_W/QALY_1
replace NHSCOSTW_Q1=0 if NHSCOSTW_Q1==.
generate TCOSTW_Q1 = tCOST_W/QALY_1
replace TCOSTW_Q1=0 if TCOSTW_Q1==.
label variable NHSCOSTW_Q1 "Total costs/QALY NHS perspective W/O hospitalization costs"
label variable TCOSTW_Q1 "Total costs/QALY  W/O hospitalization costs"
format %9.2f TCOSTW_Q1
format %9.2f NHSCOSTW_Q1
ttest NHSCOSTW_Q1, by(ARM_N)
ttest TCOSTW_Q1, by(ARM_N)
```

**** SUPPLEMENTAL ANALYSIS (TABLE S2.3. MEAN COSTS PER PATIENT-YEAR AND COST-UTILITY ANALYSIS, WITHOUT HOSPITALIZATION)***

```
heabs tCOST_NHS_W QALY_1, res(bene) int(ARM_N) w2p (30000)
heabs tCOST_NHS_W QALY_1, res(bene) int(ARM_N) w2p (50000)
heabs tCOST_W QALY_1, res(bene) int(ARM_N) w2p (30000)
heabs tCOST_W QALY_1, res(bene) int(ARM_N) w2p (50000)
```

**** SUPPLEMENTAL ANALYSIS (TABLE S3.1. DISTRIBUTION OF THE MOST RELEVANT VARIABLES BY GROUP: MEDIAN, MIN, P25, P75 AND MAX).***

***** In-office visits, patient/year***

```
by ARM_N, sort: summarize n_HOSP_TRANSM, detail
```

***** Pacemaker transmission, patient/year***

```
by ARM_N, sort: summarize t_TRANSM, detail
```

***** Physician time, min/patient***

```
by ARM_N, sort: summarize PHYS_MIN, detail
** Hospitalization days
by ARM_N, sort: summarize HOSP_DAYS, detail
** Distance home/hosp (Km)
by ARM_N, sort: summarize DISTANCE, detail
** Patients' time (travel & visits)
by ARM_N, sort: summarize PATIENT_TIME, detail

** Physician costs
by ARM_N, sort: summarize COST_DR, detail
** Consultation room costs
by ARM_N, sort: summarize COST_ROOM, detail
** Hospitalization days costs
by ARM_N, sort: summarize COST_HOSP, detail
** Ambulance transport costs
by ARM_N, sort: summarize COST_AMBUL, detail
** Total NHS costs
by ARM_N, sort: summarize tCOSTS_NHS, detail

** Patient travel costs
by ARM_N, sort: summarize COST_P_TRAVEL, detail
by ARM_N, sort: summarize COST_P_TRAV_WAIT, detail
** Other transport costs
by ARM_N, sort: summarize COST_P_OTH_TRAV, detail
** Accompanying person costs
by ARM_N, sort: summarize COST_P_ACOMP, detail
by ARM_N, sort: summarize HOSP_CONS, detail
** Total patient costs
by ARM_N, sort: summarize tCOST_PATIENT, detail

** Total (NHS + patient family) costs
by ARM_N, sort: summarize tCOSTS, detail

** Total NHS costs and total costs W/O hospitalization costs
by ARM_N, sort: summarize tCOST_NHS_W, detail
by ARM_N, sort: summarize tCOST_W, detail

** EQ5D utilities and QALYs
by ARM_N, sort: summarize EQ5D_MONTH_0, detail
by ARM_N, sort: summarize EQ5D_MONTH_1, detail
by ARM_N, sort: summarize EQ5D_MONTH_6, detail
by ARM_N, sort: summarize EQ5D_MONTH_12, detail
by ARM_N, sort: summarize QALY_1, detail

** Cost per QALY
by ARM_N, sort: summarize NHSCOST_Q1, detail
```

```
by ARM_N, sort: summarize TCOST_Q1, detail
by ARM_N, sort: summarize NHSCOSTW_Q1, detail
by ARM_N, sort: summarize TCOSTW_Q1, detail
```

```
* DO STATA FILE. NORLAND COST-UTILITY STUDY

* PATIENTSÕ CLINICAL CHARACTERISTICS AT BASELINE (TABLE 1)
** ÒsummarizeÓ command (for describing) and ÒttestÓ command (for
differences between groups) for quantitative variables, were used, and
ÒtabÓ command with chi square or FisherÕs exact test for qualitative
variables.

** Age and sex.
by ARM_N, sort: summarize AGE, detail
ttest AGE, by(ARM_N)
tab SEX ARM_N, row col chi exact
prtest SEX, by(ARM_N)

** Pacing indication, symptoms and stimulation.
tab ORIGIN ARM_N, row col chi exact
tab INDICATION ARM_N, row col chi exact
tab SYMPTOMS ARM_N, row col chi exact
tab STIMULATION ARM_N, row col chi exact

** Comorbidities:  high blood pressure, coronary heart disease, diabetes,
tachyarrhythmia, obesity (BMI>30), other, none.
tab C_HBP ARM_N, row col chi exact
tab C_CHD ARM_N, row col chi exact
tab C_TACHYARRHYT ARM_N, row col chi exact
tab C_DISLYP ARM_N, row col chi exact
tab C_DM ARM_N, row col chi exact
tab C_OBESI ARM_N, row col chi exact
tab C_OTHER2 ARM_N, row col chi exact
tab C_NONE ARM_N, row col chi exact

** Treatment (basal): antiplatelet agents, oral anticoagulants, anti-
arrhythmic drugs, anti-hypertensive drugs.
tab TR_PLAQ ARM_N, row col chi exact
tab TR_OAC ARM_N, row col chi exact
tab TR_ARRIT ARM_N, row col chi exact
tab TR_ANTIHT ARM_N, row col chi exact

** EQ5D basal (month 0) and at first month.
by ARM_N, sort: summarize EQ5D_MONTH_0, detail
ttest EQ5D_MONTH_0, by(ARM_N)
by ARM_N, sort: summarize EQ5D_MONTH_1, detail
ttest EQ5D_MONTH_1, by(ARM_N)


* COST INPUTS, COSTS PER PATIENT YEAR, AND QUALITY OF LIFE OUTCOMES
(TABLE 2)
** ÒttestÓ command for differences between groups was used.

** UTILIZATION
** In-office visits, patient/year
ttest n_HOSP_TRANSM, by(ARM_N)
** Pacemaker transmission, patient/year
ttest t_TRANSM, by(ARM_N)
** Physician time, min/patient
ttest PHYS_MIN, by(ARM_N)
** Hospitalization days
ttest HOSP_DAYS, by(ARM_N)
```

```
** Distance home/hosp (Km)
ttest DISTANCE, by(ARM_N)
** PatientsÕ time (travel & visits)
ttest PATIENT_TIME, by(ARM_N)

** NHS COSTS
** Physician costs
ttest COST_DR, by(ARM_N)
** Consultation room costs
ttest COST_ROOM, by(ARM_N)
** Hospitalization costs
ttest COST_HOSP, by(ARM_N)
** Ambulance transport costs
ttest COST_AMBUL, by(ARM_N)
** Total NHS costs
generate tCOSTS_NHS = COST_DR + COST_ROOM + COST_HOSP + COST_AMBUL
label variable tCOSTS_NHS "Total costs NHS perspective"
format %9.2f tCOSTS_NHS
ttest tCOSTS_NHS, by(ARM_N)

** PATIENT COSTS
** Patient travel costs
ttest COST_P_TRAVEL, by(ARM_N)
** Patient travel+waiting costs
ttest COST_P_TRAV_WAIT, by(ARM_N)
** Other transport costs
ttest COST_P_OTH_TRAV, by(ARM_N)
** Accompanying person costs
ttest COST_P_ACOMP, by(ARM_N)
ttest HOSP_CONS, by(ARM_N)
** Total patient costs
generate tCOST_PATIENT = COST_P_TRAV_WAIT + COST_P_OTH_TRAV +
COST_P_ACOMP
label variable tCOST_PATIENT "Total patient&family costs"
format %9.2f tCOST_PATIENT
ttest tCOST_PATIENT, by(ARM_N)

** TOTAL (NHS + PATIENT&FAMILY) COSTS
generate tCOSTS = tCOSTS_NHS + tCOST_PATIENT
label variable tCOSTS "Total costs (NHS+Patients&family)"
ttest tCOSTS, by(ARM_N)

** OUTCOMES
** Hospitalizations and deaths
by ARM_N, sort: ci HOSPITALIZ, binomial total
prtest HOSPITALIZ, by(ARM_N)
by ARM_N, sort: ci DEATH, binomial total
prtest DEATH, by(ARM_N)
** Quality of life at 6 and 12 months
ttest EQ5D_MONTH_6, by(ARM_N)
by ARM_N, sort: ci HOSPITALIZ, binomial total
ttest EQ5D_MONTH_12, by(ARM_N)

** Generating and analyzing QALYs
** Generating QALY1 as lineal interpolation between EQ5D weights at month
1 and 6, between month 6 and 12, and between the two resultant values.
generate QALY_1 = (((EQ5D_MONTH_1 + EQ5D_MONTH_6)/2) + ((EQ5D_MONTH_6 +
EQ5D_MONTH_12)/2))/2
```

```
label variable QALY_1 "QALYs"
format %9.4f QALY_1
ttest QALY_1, by(ARM_N)

* COSTS PER PATIENT/YEAR AND COSTĐUTILITY ANALYSIS (TABLE 3)
** Generating cost per QALY variables and testing differences.
generate NHSCOST_Q1 = tCOSTS_NHS/QALY_1
replace NHSCOST_Q1=0 if NHSCOST_Q1==.
generate TCOST_Q1 = tCOSTS/QALY_1
replace TCOST_Q1=0 if TCOST_Q1==.
label variable NHSCOST_Q1 "Cost/QALY NHS perspective"
format %9.2f NHSCOST_Q1
label variable TCOST_Q1 "Total Cost/QALY"
format %9.2f TCOST_Q1
ttest NHSCOST_Q1, by(ARM_N)
ttest TCOST_Q1, by(ARM_N)

** Cost-utility analysis (total NHS costs and total costs) using heabs
stata command (with a WTP threshold of 30000 and 50000 euros).
heabs tCOSTS_NHS QALY_1, res(bene) int(ARM_N) w2p (30000)
heabs tCOSTS_NHS QALY_1, res(bene) int(ARM_N) w2p (50000)
heabs tCOSTS QALY_1, res(bene) int(ARM_N) w2p (30000)
heabs tCOSTS QALY_1, res(bene) int(ARM_N) w2p (50000)

* The probabilistic analysis was carried out with the HDS online
calculator (http://www.healthstrategy.com/evpiinb/evpiinb01.htm)

* SUPPLEMENTAL ANALYSIS (TABLE S2.2. COSTS PER PATIENT-YEAR EXCLUDING
HOSPITALIZATION DAYS COSTS)
generate tCOST_NHS_W = tCOSTS_NHS - COST_HOSP
generate tCOST_W = tCOSTS - COST_HOSP
label variable tCOST_NHS_W "Total costs NHS perspective W/O
hospitalization costs"
label variable tCOST_W "Total costs W/O hospitalization costs"
format %9.2f tCOST_NHS_W
format %9.2f tCOST_W
ttest tCOST_NHS_W, by(ARM_N)
ttest tCOST_W, by(ARM_N)

** Generating cost (w/o hospitalization days) per QALY variables.
generate NHSCOSTW_Q1 = tCOST_NHS_W/QALY_1
replace NHSCOSTW_Q1=0 if NHSCOSTW_Q1==.
generate TCOSTW_Q1 = tCOST_W/QALY_1
replace TCOSTW_Q1=0 if TCOSTW_Q1==.
label variable NHSCOSTW_Q1 "Total costs/QALY NHS perspective W/O
hospitalization costs"
label variable TCOSTW_Q1 "Total costs/QALY  W/O hospitalization costs"
format %9.2f TCOSTW_Q1
format %9.2f NHSCOSTW_Q1
ttest NHSCOSTW_Q1, by(ARM_N)
ttest TCOSTW_Q1, by(ARM_N)


* SUPPLEMENTAL ANALYSIS (TABLE S2.3. MEAN COSTS PER PATIENT-YEAR AND
COST-UTILITY ANALYSIS, WITHOUT HOSPITALIZATION)
heabs tCOST_NHS_W QALY_1, res(bene) int(ARM_N) w2p (30000)
heabs tCOST_NHS_W QALY_1, res(bene) int(ARM_N) w2p (50000)
heabs tCOST_W QALY_1, res(bene) int(ARM_N) w2p (30000)
```

```
heabs tCOST_W QALY_1, res(bene) int(ARM_N) w2p (50000)


* SUPPLEMENTAL ANALYSIS (TABLE S3.1. DISTRIBUTION OF THE MOST RELEVANT
VARIABLES BY GROUP: MEDIAN, MIN, P25, P75 AND MAX).

** In-office visits, patient/year
by ARM_N, sort: summarize n_HOSP_TRANSM, detail
** Pacemaker transmission, patient/year
by ARM_N, sort: summarize t_TRANSM, detail
** Physician time, min/patient
by ARM_N, sort: summarize PHYS_MIN, detail
** Hospitalization days
by ARM_N, sort: summarize HOSP_DAYS, detail
** Distance home/hosp (Km)
by ARM_N, sort: summarize DISTANCE, detail
** PatientsÕ time (travel & visits)
by ARM_N, sort: summarize PATIENT_TIME, detail

** Physician costs
by ARM_N, sort: summarize COST_DR, detail
** Consultation room costs
by ARM_N, sort: summarize COST_ROOM, detail
** Hospitalization days costs
by ARM_N, sort: summarize COST_HOSP, detail
** Ambulance transport costs
by ARM_N, sort: summarize COST_AMBUL, detail
** Total NHS costs
by ARM_N, sort: summarize tCOSTS_NHS, detail

** Patient travel costs
by ARM_N, sort: summarize COST_P_TRAVEL, detail
by ARM_N, sort: summarize COST_P_TRAV_WAIT, detail
** Other transport costs
by ARM_N, sort: summarize COST_P_OTH_TRAV, detail
** Accompanying person costs
by ARM_N, sort: summarize COST_P_ACOMP, detail
by ARM_N, sort: summarize HOSP_CONS, detail
** Total patient costs
by ARM_N, sort: summarize tCOST_PATIENT, detail

** Total (NHS + patient family) costs
by ARM_N, sort: summarize tCOSTS, detail

** Total NHS costs and total costs W/O hospitalization costs
by ARM_N, sort: summarize tCOST_NHS_W, detail
by ARM_N, sort: summarize tCOST_W, detail

** EQ5D utilities and QALYs
by ARM_N, sort: summarize EQ5D_MONTH_0, detail
by ARM_N, sort: summarize EQ5D_MONTH_1, detail
by ARM_N, sort: summarize EQ5D_MONTH_6, detail
by ARM_N, sort: summarize EQ5D_MONTH_12, detail
by ARM_N, sort: summarize QALY_1, detail

** Cost per QALY
by ARM_N, sort: summarize NHSCOST_Q1, detail
by ARM_N, sort: summarize TCOST_Q1, detail
```

```
by ARM_N, sort: summarize NHSCOSTW_Q1, detail
by ARM_N, sort: summarize TCOSTW_Q1, detail
```
