# Supplementary material for: Cost–utility analysis of telemonitoring versus conventional hospital-based follow-up of patients with pacemakers. The NORDLAND randomized clinical trial
Source: PLoS One. 2020 Jan 29;15(1):e0226188. doi: 10.1371/journal.pone.0226188 (PMC6988929; doi:10.1371/journal.pone.0226188)
Supplement: S1 Table — (PDF) [file pone.0226188.s005.pdf]

S1 Table. Costs included in the economic evaluation (in euros, 2015).

Item	Cost	Source/Comments
**Healthcare costs**		
• Physician	0.79<br>(€ per minute worked)	• Account Unit, Nordland Hospital.<br>• Based on the 2015 work agreement.<br>• Overtime personnel costs were not taken into Account.
• Consultation room	0.40<br>(€ per minute of visit)	• Account Unit, Nordland Hospital.<br>• Includes electricity, heating, wáter, cleaning products, medical material, etc.<br>• Depreciations costs for instruments and hospital equipment used in both types of monitoring were not considered.
• Ambulance	300.00[1]	• Account Unit, Nordland Hospital.
**Patient/relative costs**		
• Transportation/Taxi	1.60[1]	• Account Unit, Nordland Hospital.
• Transportation/Bus	0.21[1]	
• Transportation/Plane	0.11-0.43[1]	
• Transportation/Private car	0.27[1]	
• Lost income (total time spent in every hospital visit)	15.00[1]	• Cost of home help service.<br>• Account Unit, Nordland Hospital.

[1] € per round trip
