# Supplementary material for: Cost–utility analysis of telemonitoring versus conventional hospital-based follow-up of patients with pacemakers. The NORDLAND randomized clinical trial
Source: PLoS One. 2020 Jan 29;15(1):e0226188. doi: 10.1371/journal.pone.0226188 (PMC6988929; doi:10.1371/journal.pone.0226188)
Supplement: S2 Table — (PDF) [file pone.0226188.s006.pdf]

Cost-utility analysis of telemonitoring versus conventional hospital-based follow-up of patients with pacemakers.
The NORDLAND randomized clinical trial.

STATISTICAL DISTRIBUTION OF STUDY VARIABLES

S2 Table Statistical distribution of study variables

	Telemonitoring (TM)							Conventional Monitoring (CM)					
	Mean	Median	Min	P25	P75	Max		Mean	Median	Min	P25	P75	Max
**Cost Inputs**													
In-office visits, patient/year	1.6	1.0	1.0	1.0	2.0	6.0		1.6	1.0	1.0	1.0	2.0	5.0
PM transmission, patient/year	11.5	12.0	1.0	10.0	14.0	18.0		1.6	1.0	1.0	1.0	2.0	5.0
Physician time, min/patient	96.6	90.0	30.0	75.0	105.0	230.0		46.8	30.0	30.0	30.0	60.0	150.0
Hospitalization days	2.3	0.0	0.0	0.0	0.0	34.0		0.0	0.0	0.0	0.0	0.0	0.0
Distance home/hosp., Km	93.1	60.0	1.0	19.0	100.0	500.0		56.7	50.0	1.0	6.0	70.0	300.0
Patients' time (travel & visits)	379.2	300.0	60.0	180.0	600.0	1800.0		307.2	300.0	60.0	120.0	480.0	900.0
	**Telemonitoring (TM)**							**Conventional Monitoring (CM)**					
**NHS costs (€2015)**													
Physician costs	76.4	71.2	23.7	59.3	83.0	181.9		37.0	23.7	23.7	23.7	47.4	118.6
Consultation room costs	39.1	36.4	12.1	30.4	42.5	93.1		18.9	12.1	12.1	12.1	24.3	60.7
Hospitalization costs	1808.3	0.0	0.0	0.0	0.0	26966.1		0.0	0.0	0.0	0.0	0.0	0.0
Ambulance transport costs	156.0	0.0	0.0	0.0	300	600		216.0	0.0	0.0	0.0	300	900
Total NHS costs	2079.8	335.9	83.7	113.6	407.6	27685.7		272.0	179.4	35.9	35.9	335.9	1007.6
	**Telemonitoring (TM)**							**Conventional Monitoring (CM)**					
**Patient/family costs (€2015)**													
Patient travel costs	34.2	16.6	0.0	0.0	33.2	332.2		20.3	11.1	0.0	0.0	22.1	110.7
Patient travel & waiting costs	59.9	27.7	0.0	0.0	44.3	465.2		24.9	22.1	0.0	0.0	44.3	110.7
Patient travel costs (other)	61.4	8.4	0.0	0.0	44.0	756.0		56.7	0.8	0.0	0.0	58.4	635.9
Accompanying person costs	94.8	75.0	15.0	45.0	150.0	450.0		76.8	75.0	15.0	30.0	120.0	225.0
Total patient costs	216.1	140.5	15.42	80.1	265.0	1671.2		158.4	118.4	30.0	63.2	195.6	650.9
**Total costs**													
Total (NHS + patient & family) costs	2295.9	446.7	129.0	252.0	560.2	27868.9		430.4	373.9	73.4	169.6	477.3	1232.6
Total NHS costs w/o hospitalization	271.5	233.2	83.7	107.6	389.7	725.6		272.0	179.4	35.9	35.9	335.9	1007.6
Total costs w/o hospitalization	487.6	429.0	129.0	252.0	557.8	1946.2		430.4	373.9	73.4	169.6	477.3	1232.6

S2 Table. Statistical distribution of study variables

	Telemonitoring (TM)						Conventional Monitoring (CM)					
	**Mean**	**Median**	**Min**	**P25**	**P75**	**Max**	**Mean**	**Median**	**Min**	**P25**	**P75**	**Max**
**Quality of life outcomes**												
EQ5D-3L at month 0	0.754	0.825	-0.011	0.710	1.000	1.000	0.813	0.799	0.514	0.719	0.914	1.000
EQ5D-3L at month 1	0.761	0.816	0.000	0.737	0.914	1.000	0.822	0.887	0.256	0.719	1.000	1.000
EQ5D-3L at month 6	0.816	0.905	0.000	0.843	1.000	1.000	0.691	0.799	-0.031	0.648	0.887	1.000
EQ5D-3L at month 12	0.729	0.816	0.000	0.710	0.905	1.000	0.783	0.887	0.000	0.710	1.000	1.000
QALYs	0.780	0.855	0.000	0.783	0.902	0.972	0.746	0.855	0.195	0.687	0.899	0.972
	**Telemonitoring (TM)**						**Conventional Monitoring (CM)**					
**Cost per QALY (€ 2015)**												
Cost per QALY (NHS perspective)	2623.6	418.7	0.00	135.3	517.3	37463.7	416.8	239.5	37.5	72.3	691.3	1720.2
Cost per QALY (total)	2874.9	548.1	0.00	259.2	871.2	37711.6	742.9	494.5	90.9	389.0	732.1	5053.8
Cost per QALY (NHS) W/O hospitalization	382.4	262.0	0.00	110.3	446.8	1707.4	416.8	239.5	37.5	72.3	691.3	1720.2
Cost per QALY (total) W/O hospitalization	633.7	521.4	0.00	259.2	766.8	2207.9	742.9	494.5	90.9	389.0	732.1	5053.8

PM: Pacemaker; NHS: National Health Service; EQ5D-3L: EuroQol 5 Dimension 3L questionnaire; QALY: Quality Adjusted Life Year.
