# Supplementary material for: Cost–utility analysis of telemonitoring versus conventional hospital-based follow-up of patients with pacemakers. The NORDLAND randomized clinical trial
Source: PLoS One. 2020 Jan 29;15(1):e0226188. doi: 10.1371/journal.pone.0226188 (PMC6988929; doi:10.1371/journal.pone.0226188)
Supplement: S3 Table — (PDF) [file pone.0226188.s007.pdf]

S3 Table. Costs per patient-year (excluding hospitalization days costs).

	All (n=50)	Groups TM (n=25)	CM (n=25)	*p*
NHS costs (€ 2015), mean (95%CI)				
Total	271.75 (205.27; 338.22)	271.53 (193.52; 349.53)	271.97 (158.18; 385.76)	0.995
Total (NHS + patient relative) costs (€ 2015), mean (95%CI)				
Total	458.99 (363.75; 554.24)	487.59 (336.52; 638.67)	430.39 (304.07; 556.70)	0.566

TM: Telemonitoring group; CM: Conventional Monitoring group; SD: Standard Deviation; NHS: Norwegian Healthcare System.
