# Supplementary material for: Cost–utility analysis of telemonitoring versus conventional hospital-based follow-up of patients with pacemakers. The NORDLAND randomized clinical trial
Source: PLoS One. 2020 Jan 29;15(1):e0226188. doi: 10.1371/journal.pone.0226188 (PMC6988929; doi:10.1371/journal.pone.0226188)
Supplement: S4 Table — (PDF) [file pone.0226188.s008.pdf]

S4 Table. Incremental costs per QALY (cost-utility analysis) of Telemonitoring vs. Conventional Monitoring (excluding hospitalization days costs).

Mean costs per QALY				
		Groups		
	All (n=50)	***TM (n=25)***	***CM (n=25)***	***p***
NHS costs per QALY (€)	399.60 (281.06; 518.15)	382.36 (226.08; 538.63)	416.85 (227.12; 606.59)	0.773
Total costs per QALY (€)	688.25 (467.66; 908.86)	633.65 (419.91; 847.40)	742.86 (339.30; 1146.41)	0.624

Incremental costs per QALY (cost–utility analysis) of telemonitoring vs. conventional monitoring		
Perspective	***NHS costs***	***Total costs***
Incremental costs per patient	34.50 (-204.97;273.96)	109.20 (-335.67; 554.08)
Incremental QALYs per patient	0.0339 (-0.0937;0.1615)	0.0339 (-0.0937;0.1615)
Mean ICER (€)	Dominated	1688.07
Mean INB(WTP30000)(€)	1017.14 (-2823.42; 4857.72)	959.49 (-2872.77; 4795.90)
Mean INB(WTP50000)(€)	1694.94 (-4697.60; 8087.48)	1637.29 (-4745.96; 8024.69)

TM: Telemonitoring group; CM: Conventional monitoring group; QALY: Quality-adjusted life year; NHS: National Health System; ICER: Incremental cost–effectiveness ratio; INB: Incremental net benefit; WTP: Willingness to pay.
