# Supplementary material for: Cost–utility analysis of telemonitoring versus conventional hospital-based follow-up of patients with pacemakers. The NORDLAND randomized clinical trial
Source: PLoS One. 2020 Jan 29;15(1):e0226188. doi: 10.1371/journal.pone.0226188 (PMC6988929; doi:10.1371/journal.pone.0226188)
Supplement: S3 Fig — (PDF) [file pone.0226188.s011.pdf]

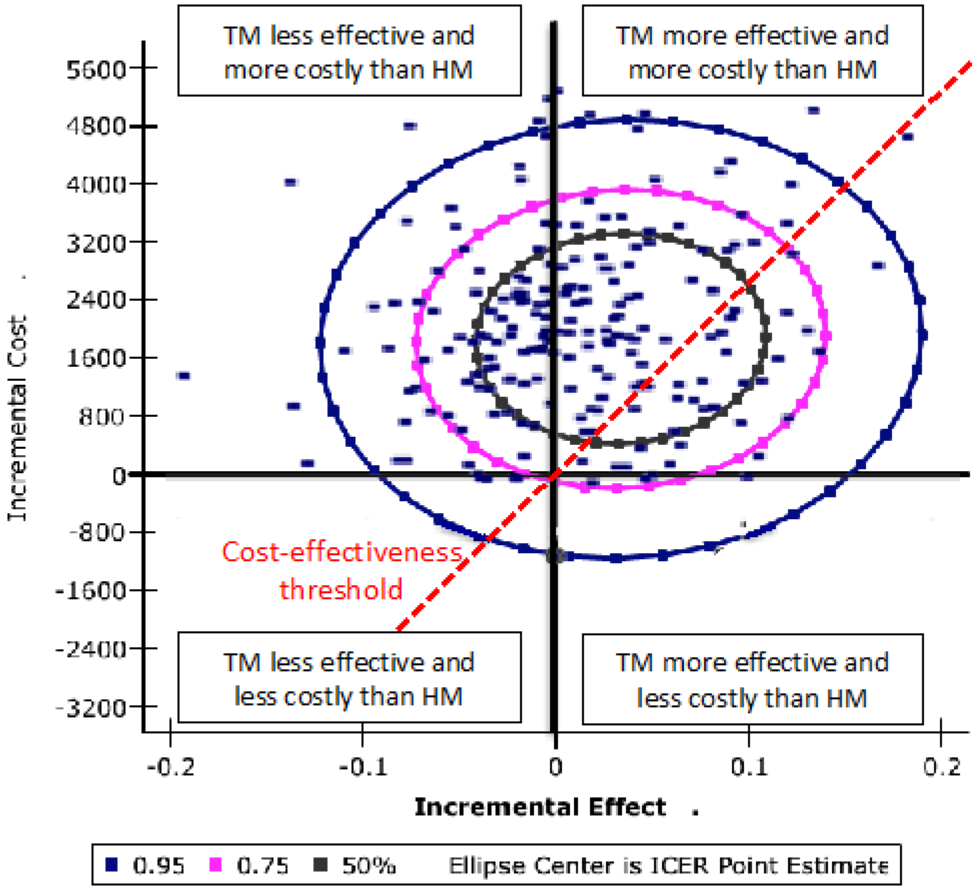

S3 Fig. Incremental Net Benefit (INB) curve at different WTP thresholds with 95% CI. NHS: National Health Service; TM: Telemonitoring; CM: Conventional monitoring group; QALY: Quality-adjusted life years; ICER: Incremental cost–effectiveness ratio; WTP: Willingness to pay.

The area to the right of the threshold in the cost-effectiveness plane includes the cost-effective ICERs generated by the bootstrapping process (to the left are the non cost-effective ones.
