# Supplementary material for: Cost–utility analysis of telemonitoring versus conventional hospital-based follow-up of patients with pacemakers. The NORDLAND randomized clinical trial
Source: PLoS One. 2020 Jan 29;15(1):e0226188. doi: 10.1371/journal.pone.0226188 (PMC6988929; doi:10.1371/journal.pone.0226188)
Supplement: S4 Fig — (PDF) [file pone.0226188.s012.pdf]

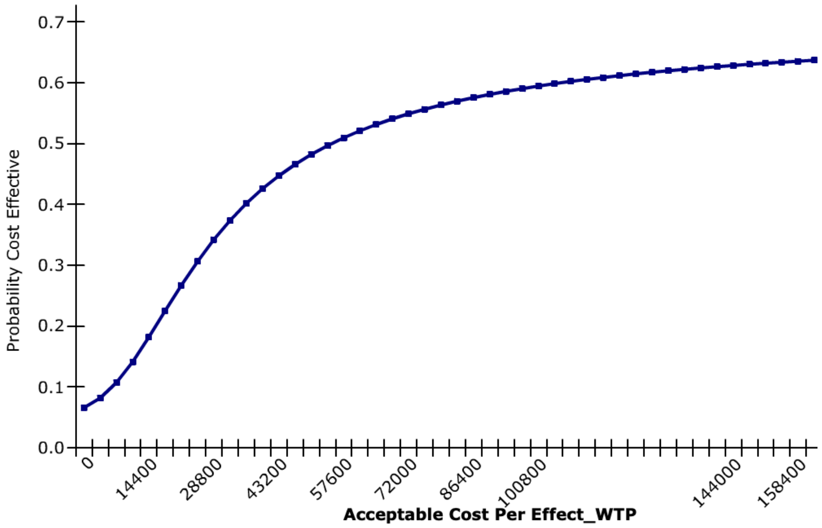

S4 Fig. Cost–Effectiveness Acceptability Curve at different WTP thresholds.
TM: Telemonitoring; HM: Hospital monitoring; QALY: Quality-adjusted life years; ICER: Incremental cost–effectiveness ratio; WTP: Willingness to pay.
INBs greater than zero shows cost-effective estimates for the respective WTP threshold.
