# Supplementary figures and images for: Cost–utility analysis of telemonitoring versus conventional hospital-based follow-up of patients with pacemakers. The NORDLAND randomized clinical trial
Source: PLoS One. 2020 Jan 29;15(1):e0226188. doi: 10.1371/journal.pone.0226188 (PMC6988929; doi:10.1371/journal.pone.0226188)

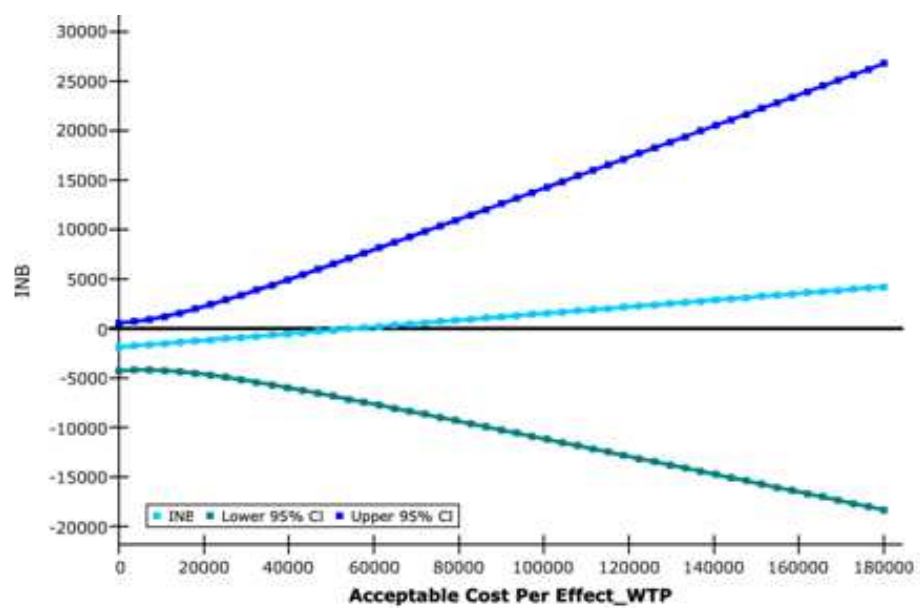

**S5 Fig. Incremental Net Benefit curve at different WTP thresholds with 95% CI.**

Supplement: S5 Fig — (PDF) [file pone.0226188.s013.pdf]
